# Forest Plant and Bird Communities in the Lau Group, Fiji

**DOI:** 10.1371/journal.pone.0015685

**Published:** 2010-12-29

**Authors:** Janet Franklin, David W. Steadman

**Affiliations:** 1 School of Geographical Sciences and Urban Planning and School of Life Sciences, Arizona State University, Tempe, Arizona, United States of America; 2 Florida Museum of Natural History, University of Florida, Gainesville, Florida, United States of America; Institut Mediterrani d'Estudis Avançats (CSIC/UIB), Spain

## Abstract

**Background:**

We examined species composition of forest and bird communities in relation to environmental and human disturbance gradients on Lakeba (55.9 km^2^), Nayau (18.4 km^2^), and Aiwa Levu (1.2 km^2^), islands in the Lau Group of Fiji, West Polynesia. The unique avifauna of West Polynesia (Fiji, Tonga, Samoa) has been subjected to prehistoric human-caused extinctions but little was previously known about this topic in the Lau Group. We expected that the degree of human disturbance would be a strong determinant of tree species composition and habitat quality for surviving landbirds, while island area would be unrelated to bird diversity.

**Methodology/Principal Findings:**

All trees >5 cm diameter were measured and identified in 23 forest plots of 500 m^2^ each. We recognized four forest species assemblages differentiated by composition and structure: coastal forest, dominated by widely distributed species, and three forest types with differences related more to disturbance history (stages of secondary succession following clearing or selective logging) than to environmental gradients (elevation, slope, rockiness). Our point counts (73 locations in 1 or 2 seasons) recorded 18 of the 24 species of landbirds that exist on the three islands. The relative abundance and species richness of birds were greatest in the forested habitats least disturbed by people. These differences were due mostly to increased numbers of columbid frugivores and passerine insectivores in forests on Lakeba and Aiwa Levu. Considering only forested habitats, the relative abundance and species richness of birds were greater on the small but completely forested (and uninhabited) island of Aiwa Levu than on the much larger island of Lakeba.

**Conclusions/Significance:**

Forest disturbance history is more important than island area in structuring both tree and landbird communities on remote Pacific islands. Even very small islands may be suitable for conservation reserves if they are protected from human disturbance.

## Introduction

The 25,000+ islands in the tropical Pacific Ocean represent discrete terrestrial habitats that vary greatly in size, isolation, origin, and age [1], [2]. Not surprisingly, the region has major geographical gaps in quantitative studies of the terrestrial biotic communities [1], [3]. This paper addresses one of those gaps in West Polynesia (WP), a region that includes Fiji, Tonga, Samoa, and the outlying islands of Rotuma, Wallis & Futuna, and Niue. People have occupied WP for nearly 3000 years [4], [5], resulting in the prehistoric reduction of forest cover [6], [7], [8], the introduction of non-native plants and animals [9], and the loss of populations (extirpations) and species (extinctions) of reptiles, bats, and birds [1], [9], [10], [11]. These impacts must be considered when interpreting the modern diversity and distribution of plants and animals, and thus are informative in conservation planning and management [12], [13], [14].

**Figure 1:**
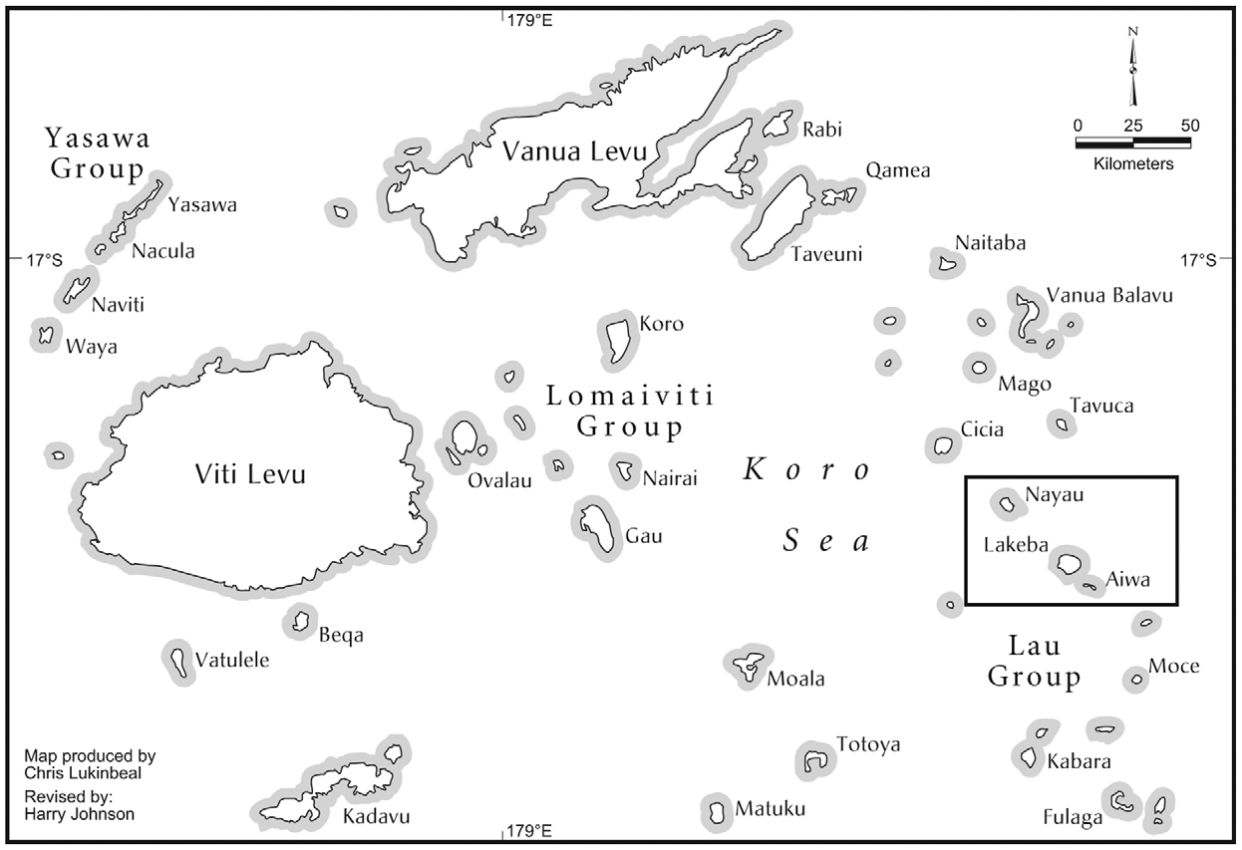
Map of Fiji. Box shows location of the islands surveyed in Central Lau.

This paper concerns the Lau Group in Eastern Fiji (Figure 1), which lies between the much larger and geologically older Fijian islands 100–200 km to the west and the more remote Tongan islands 300–350 km to the east. Excluding the geologically unrelated, eroded volcanic islands of Moala, Totoya, and Matuku, the Lau Group consists of 54 named islands (35 of them >1 km^2^) totaling 376 km^2^ of land. The plant communities of the Lau Group have been described qualitatively [e.g.], [ 15,16], although no quantitative forest composition data have previously been reported. Birds of the Lau Group have been, until recently, perhaps the most poorly known in WP. Mentioned in a very general way in Watling [17], [18], Clunie [19], and Pratt et al. [20], the relative abundance and habitat relationships were unstudied until our first point-count surveys on Lakeba in 1999 [1], [21]. During 2000 and 2001, we made additional point counts on Lakeba, and on Nayau and Aiwa Levu, allowing us to consider variation in bird community abundance and composition among seasons, islands and habitats.

Quantitative descriptions of WP forest communities exist for Tonga [22], [23], [24], [25], Samoa [26], [27], [28], [29], and western Fiji [30], [31], and in a few cases have been related to habitat quality for vertebrates [32], [33], [34]. In this paper we examine variation in species composition of woody plant and bird communities in forests on the Lau islands of Lakeba, Nayau, and Aiwa Levu. We analyze tree abundance data from forest plots to characterize variation in native forest habitats in relation to topographic and human disturbance gradients, examine differences in landbird abundance and community composition among tree-dominated habitats (including pine plantation and agroforestry), islands, and seasons, and then compare our results with those from similar studies elsewhere in WP and other tropical forest communities.

Because prehistoric and historic deforestation has been a major driver of vertebrate extirpations and extinctions in WP [1], we expected that the human disturbance gradient (agroforestry, plantation forestry, and native forest at varying stages of secondary succession) would be a strong determinant of habitat quality for landbirds [32], [33], [35] as well as tree species composition [22]. Those previous studies found that on oceanic islands with long histories of human impact, the composition of ecological communities varies to a greater degree as a function of the intensity of human disturbance than it does along environmental gradients (substrate, topography), especially when those environmental gradients are short.

We also expected, based on our previous findings in Tonga [36] and other archipelagos [1], that island area might not be positively related to bird species richness over the range of island sizes considered. A null model for species-area relationships of landbirds in Oceania [1] states that every species known to inhabit an island group should occur on each high island (non-atoll) of a certain minimum land area within that archipelago, and the findings in [36] were consistent with this null model. As noted in [36] and references therein, nonsignificant correlations between species number and area may be underreported in the literature, although they would be as informative about species-area relationships as positive correlations.

## Methods

### Study Area

Lakeba (18°14′ S, 178°48′ W; 55.9 km^2^; Figure 1) has an andesitic volcanic center of Miocene age [37] that is over 200 m maximum elevation, with uplifted limestone exposed on its western (leeward) side that covers ca. 4% of the island [16]. Deforestation for subsistence agriculture probably began soon after people first arrived about 2800 years ago [38]. As a result, nearly all forest in the volcanic uplands has been replaced by *talasiga* (a fire-degraded, herbaceous and shrubby community dominated by ferns and grasses) or, in recent decades, by pine plantations [15], [16]. Remnant native forest covers ca. 10% (<6 km^2^) of Lakeba in patches on coastal limestone outcrops and in small ravines on the volcanic hillsides [16], [39].

Nayau (17°58′ S, 179°03′ W; 18.4 km^2^) is 28 km NW of Lakeba (Figure 1). Also a composite of exposed volcanic rock and weathered limestone [37], Nayau has the “wedding cake” profile of many raised limestone islands [2]. The central plateau is a shallow depression (<100 m elevation) with cultivated volcanic soils [40]. This plateau is surrounded by a discontinuous ring of tiered, uplifted limestone (160 m maximum elevation) that sustains most of the remaining forest on Nayau.

Lying at 18°19′ S, 178°42′ W (12 km SSE of Lakeba) are the twin islets of Aiwa Levu (1.2 km^2^, ∼50 m elevation) and Aiwa Lailai (∼1 km^2^, ∼30 m elevation). They are composed entirely of limestone, surrounded by extensive reefs, and separated from each other by a narrow (∼100 m), shallow passage [41]. These islets are currently uninhabited and completely forested, although the forest understory is very disturbed by goats, with likely impacts on forest regeneration [42]. We were able to survey the vegetation and birds on Aiwa Levu but not Aiwa Lailai.

With slightly more than 2000 mm mean average rainfall at Tubou village, Lakeba, the climate in this part of Lau is transitional between “tropical wet” and “wet seasonal” [43]. Mean monthly rainfall is <100 mm only for the three winter months (June-August), with large interannual variation [16]; the forests are dominated by evergreen rather than deciduous species [e.g.], [ 30,44]. Tracking the rainfall pattern, the “small island” forests [3] of Lau are transitional in structure and composition between tropical rain forest and tropical dry forest. Of the 449 species of plants recorded thus far from Lakeba, Nayau, and Aiwa Levu, 161 (36%) are introduced and 41 (14% of the 288 indigenous species) are endemic to Fiji [39]. In this study we quantified the compositional variation of native forest (the numbered groups in Table 1), and then examined the landbird communities in tree-dominated habitats including successional stages of native forest and semi-natural and cultural (agroforestry, plantation forestry) forests, as in our preliminary study [21].

**Table 1:** Tree-dominated habitat types described for Lau.

Garnock-Jones [15]	Latham & Brookfield [16]	Franklin et al. [39]	Steadman & Franklin [21]	This Study (Group)
Plantations and Gardens	Coconut Plantation	Cultural Vegetation	Coastal Coconut Plantation (agriculture)	Coastal Coconut Plantation
--	--	Cultural Vegetation	Pure Pine Woodland (exotic *Pinus caribaea* plantation)	Pure Pine Woodland
--	--	Cultural Vegetation	Mixed Pine Woodland (pine plantation with regenerating native forest)	Mixed Pine Woodland
--	--	Coastal Forest	--	Coastal Forest (1)
Young Forest	--	Lowland Limestone Forest	Native Lowland Forest on Limestone (secondary)	Secondary Forest (2)
Mature Forest	Evergreen Ombrophilous Forest on Limestone	Lowland Limestone Forest	Native Lowland Forest on Limestone (mid/late-successional)	Mid-successional Forest (3, 4)

For native forest vegetation analyzed quantitatively in this study, group numbers are given in parentheses.

The modern avifauna of the Lau Group is dominated by species that are widespread in WP. The Lau Group lacks most of the species of landbirds found on the six largest, western Fijian islands. Only two (*Mayrornis lessoni*, *Myzomela jugularis*) of the 21 species that occur on Lakeba, the largest island in Lau and the tenth largest in Fiji, are endemic to Fiji. Of the 24 species of landbirds that inhabit the entire Lau Group today, the only one endemic to Lau is *Mayrornis versicolor*, a monarch confined to Ogea Levu [1: Table 6–5].

### Forest Community Data and Analysis

In 1999–2001, we established 23 vegetation plots in coastal and lowland forests [sensu 39], including 12 on Lakeba, seven on Nayau, and four on Aiwa Levu. Although vegetation sampling was not even among islands, it was roughly proportional to their forested area. The plots on Lakeba were located in two main areas of coastal limestone outcrops – Tarakua (SE of Tubou village) and Vagadra (SW of Nasaqalau village). The plots on Nayau were located on limestone escarpments on the eastern, southern, and western sides of the island, in the vicinities of the three villages. The plots on Aiwa Levu were located in the central and eastern portions of the island. Difficulties with logistics of travel, accommodation, and health limited our data collection in these remote islands.

Plot locations reflected gradients of human disturbance (different distances from habitation and cultivation) and environment (with varying slopes, rockiness and elevations). The plot size of 500 m^2^ (10×50 m) is adequate to capture patterns of tree species composition in small forest patches, and is similar to the plot size used in forest vegetation sampling elsewhere in Polynesia [22], [29], [45]. Data collection methods were also very similar to those used in the region [24]. All tree stems with diameter at breast height (DBH; 1.3 m) >5 cm were measured and identified to species. For each plot, we also recorded: a) elevation (m) from 1∶25,000 topographic maps; b) slope aspect (degrees), the direction that the slope faces measured by compass and converted to a northness index for analysis using a cosine transform; c) slope angle (degrees), the steepness of the slope measured by clinometer; d) rockiness (% of ground covered by exposed limestone); and, e) canopy height, estimated by averaging the height of 3–5 tall trees measured using a clinometer and tape measure.

Tree species accumulation with total surveyed area was examined to estimate the extent to which the sample (23 plots) captured the forest community species pool, in order to confirm that the small sample is representative of compositional variation. An asymptotic species accumulation curve would suggest that the sample adequately described the community. Clustering [46] was applied to the species-by-plot matrix to detect groups of plots with similar species composition based on Bray-Curtis distance calculated from relative abundance (percent basal area by species), using group averaging as the linkage method [47]. The multi-response permutation procedure [MRPP; 48] tested for the significance of differences in composition among groups of plots delineated by clustering. Then, in order to interpret and describe the composition of the resulting forest groups, indicator species analysis [49] was carried out to identify those species uniquely associated with, and thus characterizing, each group. The significance of each identified indicator species' indicator values (the average of relative abundance and relative frequency) was determined by simulation.

Species and environmental data were then subjected to constrained multivariate ordination using partial Canonical Correspondence Analysis [pCCA], [ 50,51] in order to identify environmental correlates of compositional variation among plots and groups of plots. The effect of island (treated as a covariable) was first removed from the species-by-plot community matrix because a comparable range of environmental conditions did not occur on each island. Then the residual community matrix was subjected to ordination constrained by the environmental variables.

### Bird Community Data and Analysis

Our point-count methods were identical or very similar to those used elsewhere in the region [35], [52]. During 9–15 May 1999 (dry season, Lakeba), 21 February – 6 March 2000 (wet season, Lakeba and Aiwa Levu), and 23–24 October 2001 (dry season, Nayau), we recorded each bird seen or heard within a fixed 50-m radius over a five-minute period at 42 points on Lakeba, 16 points on Nayau, and 15 points on Aiwa Levu. These points were located in the six tree-dominated cultural and native habitats listed in Table 1 (last column). The point counts done on Lakeba in May 1999 [published in 21] were repeated in February-March 2000, whereas only a single count was done at each point on Nayau and Aiwa Levu. Most point counts were done jointly by DWS and JF; a few were by DWS alone. Birds detected at distances >50 m were noted but not included in the analyses. The point-count transects in anthropogenic habitats (Coastal Coconut Plantation, Pure Pine Woodland; Table 1) followed trails or dirt roads to allow rapid, quiet travel between stations. Point-count stations in native forest were not along trails; walking through this rugged limestone terrain was noisier than in other habitats, so we waited two minutes at each station in forest before beginning the count period. Because the swiftlet *Collocalia spodiopygia* vocalizes irregularly and is much easier to detect (visually) in open habitats than in forests, we do not include its data in the total relative abundance values.

Each point-count station was at least 150 m apart from any other. Each point count was done within ca. 3 hr of sunrise (0552-0857 hr on Lakeba, 0531-0734 hr on Nayau, and 0620-0834 hr on Aiwa Levu). While we report all of these data, we provide interisland comparisons only for the February-March 2000 (wet season) data of Lakeba vs. Aiwa Levu, thereby eliminating the potential effects of seasonality and interannual variability. Avian taxonomy and nomenclature follow that of Steadman (2006).

We examined the variation in species richness and abundance of landbirds by season (fixed effect) and habitat type (nested within seasons) for Lakeba using generalized linear mixed effects Poisson models (for count data). Because these estimates of species richness per habitat are biased by unequal sample sizes (point counts per habitat), we also carried out rarefaction analysis to interpolate estimated species richness to equal sample sizes. We used the function rarefy in the package vegan of the R software [53]. This function is based on Hurlbert's formulation [54], and the standard errors on Heck et al. [55].

We also compared abundances of functional (feeding) guilds of landbirds, including columbid frugivores/granivores, passerine insectivores, and nectarivores, between seasons (fixed effect) and among habitats (nested within season) on Lakeba using Poisson generalized linear mixed effects models for count data. Then we compared the species richness and abundance of forest-obligate guilds (passerine insectivores and columbids) on Lakeba vs. Aiwa Levu based only on the 2000 wet season data (collected during the same months on both islands) and only for point counts in native forest habitat (roughly equal samples and therefore not requiring rarefaction), using non-parametric Kruskal-Wallis tests.

These were the only statistical comparisons of bird data we made owing to the unbalanced sampling (over islands, habitats and time); other patterns are described qualitatively. Although the comparisons of abundances of species or guilds among habitats and islands may be affected by species-specific detectability that can vary by habitat [56], we did not estimate detectability [as in 57] given our small sample. Nevertheless, based on our double-observer approach to estimate relative abundance [see 58] and our familiarity with WP landbirds (surveys on 50+ islands in Fiji, Tonga, and Samoa), the only common species that is vocally inconspicuous is the forest is the swiftlet *Collocalia spodiopygia* which, as already mentioned, was excluded from the comparisons.

## Results

### Tree Community Composition

We recorded 85 species of trees >5 cm DBH in the 23 Lau forest plots, including eight of indeterminate identity (Supplementary Table S1). Based on the asymptotic pattern of species accumulation in the plots (Supplementary Figure S1), we concluded that the sample was reasonably adequate to describe forest composition; the estimated number of species in the sampled forests is 112-127 using first- and second-order jackknife estimates [59].

Clustering identified four groups of plots, although they overlap somewhat in species composition (Table 2). MRPP indicates, however, that the groups are distinctive (P≪0.001), having within-group homogeneity typical of plant community data (A = 0.207). Partial CCA showed that the contribution to the mean square contingency coefficient of the conditioning factor, Island, was 16% and of the constraining environmental variables was 17%. The percent contribution of the first three pCCA axes to the contingency coefficient, after removing the contribution of Island, was 7%, 7% and 3%, respectively. pCCA Axis 1 is significantly (α = 0.1) positively correlated with slope angle and negatively with elevation, Axis 2 is negatively correlated with rockiness, and Axis 3 is correlated canopy height (Table 3). However, a permutation test showed the overall effect of the environmental variables to be insignificant. These results suggest that variation in forest composition among plots is only weakly related to the measured gradients of forest structure and topography, although some differences among groups are suggested by the data (Table 4) and will be described.

**Table 2:** Basal area (BA; m^2^/ha) of selected species in groups of plots within Forest types (Table 1).

		Group 1. Coastal		Group 2. Secondary		Group 3. Mid-successional		Group 4. Mid-successional	
Tree Species	Indic.	BA	(s.d.)	BA	(s.d).	BA	(s.d.)	BA	(s.d.)
*Pisonia grandis*	Gp 1*	11.24	(12.60)	--	--	0.14	(0.38)	--	--
*Xylosma simulans*	Gp 1***	6.83	(0.48)	0.04	(0.11)	0.22	(0.47)	0.54	(0.76)
*Pouteria grayana*		4.11	(8.02)	0.14	(0.35)	1.82	(4.71)	--	--
*Maniltoa floribunda*		2.52	(2.93)	0.67	(1.32)	0.13	(0.25)	--	--
*Ficus obliqua*		1.92	(3.85)	1.30	(2.57)	--	--	--	--
*Harpullia arborea*	Gp 1*	1.83	(2.88)	--	--	0.05	(0.14)	--	--
*Tabernaemontana pandacaqui*	Gp 1***	1.69	(0.44)	0.08	(0.14)	0.02	(0.07)	--	--
*Millettia pinnata*		1.36	(2.72)	0.17	(0.44)	0.52	(0.78)	--	--
*Aglaia saltatorum*	Gp 1 *	1.25	(0.97)	0.02	(0.06)	0.11	(0.21)	--	--
*Diospyros samoensis*	Gp 1 *	1.19	(1.02)	--	--	0.06	(0.13)	0.43	(0.61)
*Guettarda speciosa*	Gp 1 *	0.60	(0.71)	0.01	(0.04)	--	--	--	--
*Diospyros elliptica*	Gp 1 *	0.16	(0.23)	--	--	--	--	--	--
*Pandanus tectorius*		0.14	(0.28)	0.38	(1.13)	--	--	--	--
*Melicope cucullata*		--	--	6.92	(18.78)	--	--	0.15	(0.21)
*Alphitonia zizyphoides*	Gp 2 ***	--	--	5.96	(4.69)	--	--	--	--
*Pleiogynium timoriense*		--	--	2.97	(5.97)	0.34	(0.96)	--	--
Lauraceae unknown sp.		--	--	1.81	(5.43)	--	--	--	--
*Macaranga seemannii*		--	--	1.70	(3.34)	--	--	--	--
*Dendrocnide harveyi*		--	--	1.48	(2.96)	--	--	0.09	(0.01)
*Neonauclea forsteri*		--	--	1.24	(3.72)	--	--	--	--
*Mangifera indica* (I)		--	--	1.11	(3.33)	--	--	--	--
*Cocos nucifera* (I)		--	--	1.07	(2.23)	0.36	(1.02)	0.67	(0.95)
*Pometia pinnata*		--	--	0.71	(2.12)	--	--	0.03	(0.04)
*Garuga floribunda*		--	--	0.69	(1.94)	--	--	--	--
*Dysoxylum richii*		4.76	(3.41)	3.77	(4.60)	8.89	(13.11)	2.29	(1.95)
*Gyrocarpus americanus*	Gp 3 *	--	--	--	--	12.68	(12.78)	0.42	(0.59)
*Dysoxylum tenuiflorum*	Gp 3 *	--	--	0.53	(0.96)	4.61	(6.47)	6.75	(9.54)
*Barringtonia edulis*		--	--	0.66	(1.08)	2.01	(3.70)	--	--
*Cryptocarya hornei*	Gp 3 *	0.13	(0.15)	0.40	(0.44)	1.61	(1.32)	--	--
*Burckella richii*		--	--	0.96	(2.00)	0.86	(1.87)	--	--
*Homalium* cf. *pallidum*		--	--	--	--	0.85	(2.40)	--	--
*Myristica gillespieana*		--	--	0.92	(2.00)	0.74	(1.98)	--	--
*Alangium vitiense*		--	--	0.61	(0.92)	0.74	(1.99)	--	--
*Elattostachys falcata*		--	--	0.19	(0.57)	0.51	(1.19)	--	--
*Polyalthia laddiana*		0.03	(0.03)	--	--	0.49	(0.910	0.16	(0.23)
*Macaranga harveyana*		--	--	0.52	(1.17)	0.17	(0.49)	--	--
*Ficus prolixa*	Gp 4 *	--	--	--	--	--	--	227.8	(233.2)
*Buchanania vitiensis*	Gp 4 *	--	--	0.69	(1.89)	0.01	(0.03)	4.77	(6.75)
*Syzygium* sp. nova	Gp 4 *	--	--	0.06	(0.18)	--	--	1.57	(2.23)
*Arytera brackenridgei*		--	--	0.01	(0.02)	0.03	(0.08)	1.52	(2.15)
*Antirhea inconspicua*		--	--	0.02	(0.04)	0.15	(0.44)	1.12	(1.58)
*Cerbera manghas*		--	--	0.41	(1.23)	--	--	0.86	(1.22)
*Vavaea amicorum*		0.03	(0.07)	0.12	(0.21)	0.41	(1.17)	0.77	(1.10)
*Polyscias multijuga*		--	--	0.08	(0.21)	0.15	(0.22)	0.45	(0.64)
*Phaleria pubiflora*	Gp 4 *	--	--	--	--	--	--	0.18	(0.26)
*Cordyline fruticosa*	Gp 4 *	--	--	--	--	--	--	0.07	(0.10)
*Syzygium* aff. *gracilipes*	Gp 4 *	--	--	--	--	--	--	0.04	(0.05)

Only species that are abundant in or indicators for at least one Group (Gp) are shown. Indicator (Indic.) species for groups,

*P<0.1;

**P<0.01;

***P<0.001. (I)  =  Introduces species (cultivated and ∼naturalized).

**Table 3:** Correlations (intra-set) of environmental variables with partial Canonical Correspondence Analysis (pCCA) ordination axes 1-3.

Variable *	pCCA 1	pCCA 2	pCCA 3
Slope angle (degrees)	0.505	0.195	0.193
Elevation (m)	-0.496	-0.287	0.238
Rockiness (% cover)	0.384	0.571	-0.241
Canopy height (m)	0.157	-0.245	0.524

**Table 4:** Mean values (and standard deviation) of environmental and forest structural characteristics of four groups of forest plots in Lau.

Variable	1. Coastal Forest		2. Secondary Forest		3. Mid-successional Forest		4. Mid-successional Forest	
N plots	4		9		8		2	
Slope angle (degrees)	2.50	(5.00)	7.78	(10.93)	6.88	(9.36)	7.50	(10.61)
cos(slope aspect)	0.93	(0.15)	0.34	(0.85)	0.51	(0.80)	0.97	(0.04)
Elevation (m)	28.75	(10.31)	79.44	(56.76)	51.88	(26.04)	40.00	(28.28)
Rockiness (% cover)	5.75	(9.60)	29.44	(33.58)	40.63	(37.84)	12.50	(17.68)
Canopy height (m)	12.75	(1.50)	18.67	(5.22)	17.00	(5.42)	14.50	(0.71)
Basal area (m^2^/ha)	40.04	(10.36)	43.77	(20.80)	40.51	(15.26)	252.06	(230.68)

**Figure 2:**
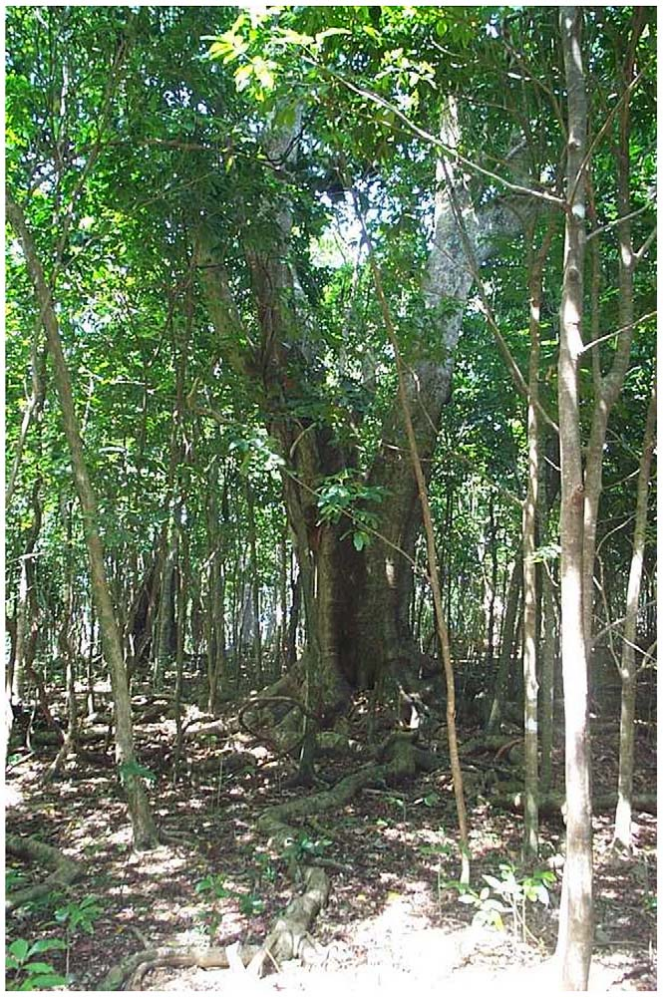
Coastal forest on Aiwa (Group 1). Large *Pisonia grandis* in center of photo. Photo by JF 11 Mar 2000.

Aiwa Levu supports a unique forest type, distinctive from those on the other two islands. Group 1 corresponds to Coastal Forest and includes four plots (all from Aiwa Levu) with shorter canopies, and occurring at lower elevations and on gentler slopes than in the other plots (Table 4). Group 1 has a diverse overstory (Table 2), with several species (*Xylosma simulans, Dysoxylum richii*, *Pouteria grayana, Maniltoa floribunda*) of large size (up to 60 cm DBH; up to 100 cm DBH for *Pisonia grandis*; Figure 2). Important smaller trees include *Aglaia saltatorum* and *Diospyros elliptica*. Because Aiwa Levu is so small (1.2 km^2^) and low (∼50 m elevation), a strong littoral (coastal) component to this forest is seen in the occurrence of species such as *Guettarda speciosa*, *Neisosperma oppositifolium*, *Pandanus tectorius*, and *Barringtonia asiatica*.

**Figure 3:**
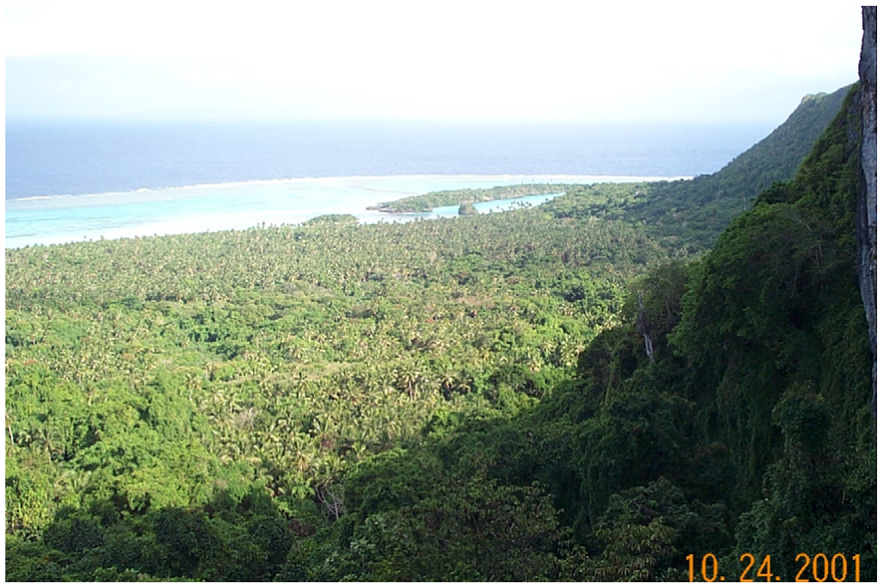
Secondary Forest on Nayau (Group 2). View of coastal plain of Nayau from escarpment at Korobuca, showing Secondary forest (smooth canopy texture) interspersed with Coastal Coconut Plantation. Photo by JF 24 Oct 2001.

Group 2, Secondary Forest (Table 1), consists of three plots from Lakeba (Tarakua) and six from Nayau (Figure 3), all dominated by *Alphitonia zizyphoides*, *Dysoxylum richii*, and *Pleiogynium timoriense*, with *Melicope cucullata* abundant only in one plot (Table 2). *Alangium vitiense*, *Cryptocarya hornei*, and *Maniltoa floribunda* were regenerating in the understory. The dominant *A. zizyphoides* is an early-successional tree, as is the less common pioneer *Macaranga* spp. [60], [61]. These plots appeared to be disturbed from selective logging, cyclones, or regeneration following cultivation based on the occurrence of tree stumps, coppicing, and cultivated tree species. Cultivated or preserved trees noted in or near Group 2 plots include *Annona muricata*, *Barringtonia edulis*, *Cocos nucifera*, *Citrus* spp., *Mangifera indica*, *Pometia pinnata*, and *Bischofia javanica*. The Secondary Forest plots have slightly greater canopy height than the other groups (Table 4); they were found on somewhat less rocky sites than the Mid-successional Forest plots (Groups 3), and therefore were more likely to have been cleared for cultivation in the past.

**Figure 4:**
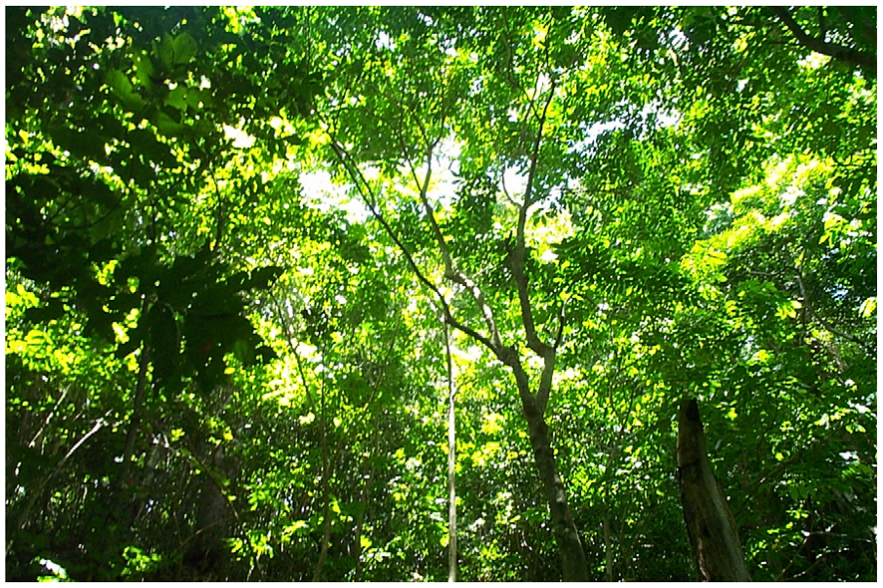
Mid-successional Forest (Group 3). View upward through *Dysoxylum*-dominated canopy at Vagadra, Lakeba. Photo by JF 7 Nov 2000.

**Figure 5:**
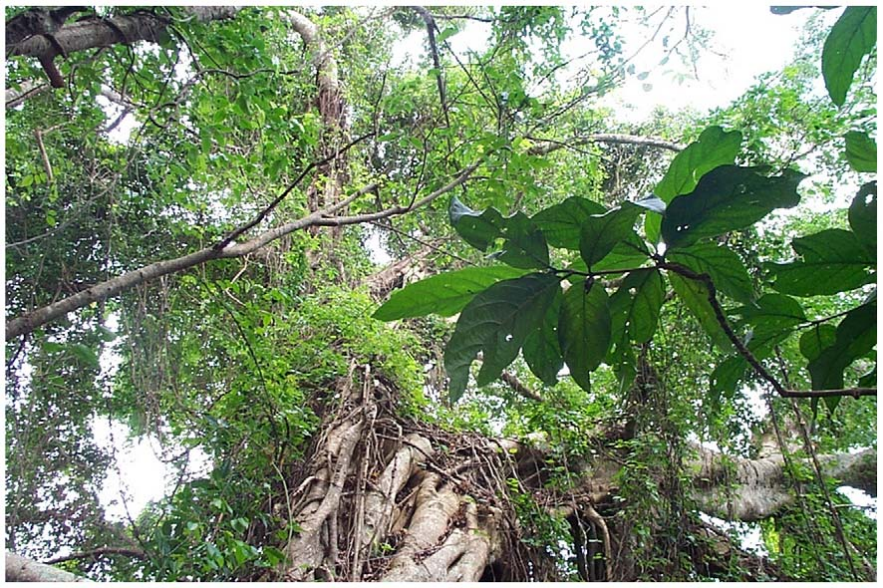
Mid-successional Forest (Group 4). View upward through canopy of a large banyan (*Ficus prolixa*) at Vagadra, Lakeba. Photo by JF 20 Feb 2000.

Group 3 represents Mid-successional Forest (Table 1) and includes seven plots on Lakeba and one on Nayau. These plots are dominated by a few very large *Gyrocarpus americanus*, with abundant *Dysoxylum richii* and/or *D. tenuiflorum* (Figure 4; Table 2). *Cryptocarya hornei* and *Pouteria grayana* are shade-tolerant species [61] that were regenerating in these plots. This forest occurs on rocky sites (Table 4). The two Group 4 plots, found in the Tarakua and Vagadra areas of Lakeba, also have abundant *D. tenuiflorum* but are distinguished from Group 3 by the presence of the large banyan, *Ficus prolixa* (Figure 5), as well as *Buchanania vitiensis* and *Phaleria pubiflora* (Table 2). Group 4 plots (also Mid-successional Forest) have shorter canopy height and extremely high basal area (owing to the banyans), and are found at somewhat lower elevations (nearer the coast), than those of Groups 2 or 3 (Table 4).

### Landbird Community Composition, Habitat Association, and Abundance

**Figure 6:**
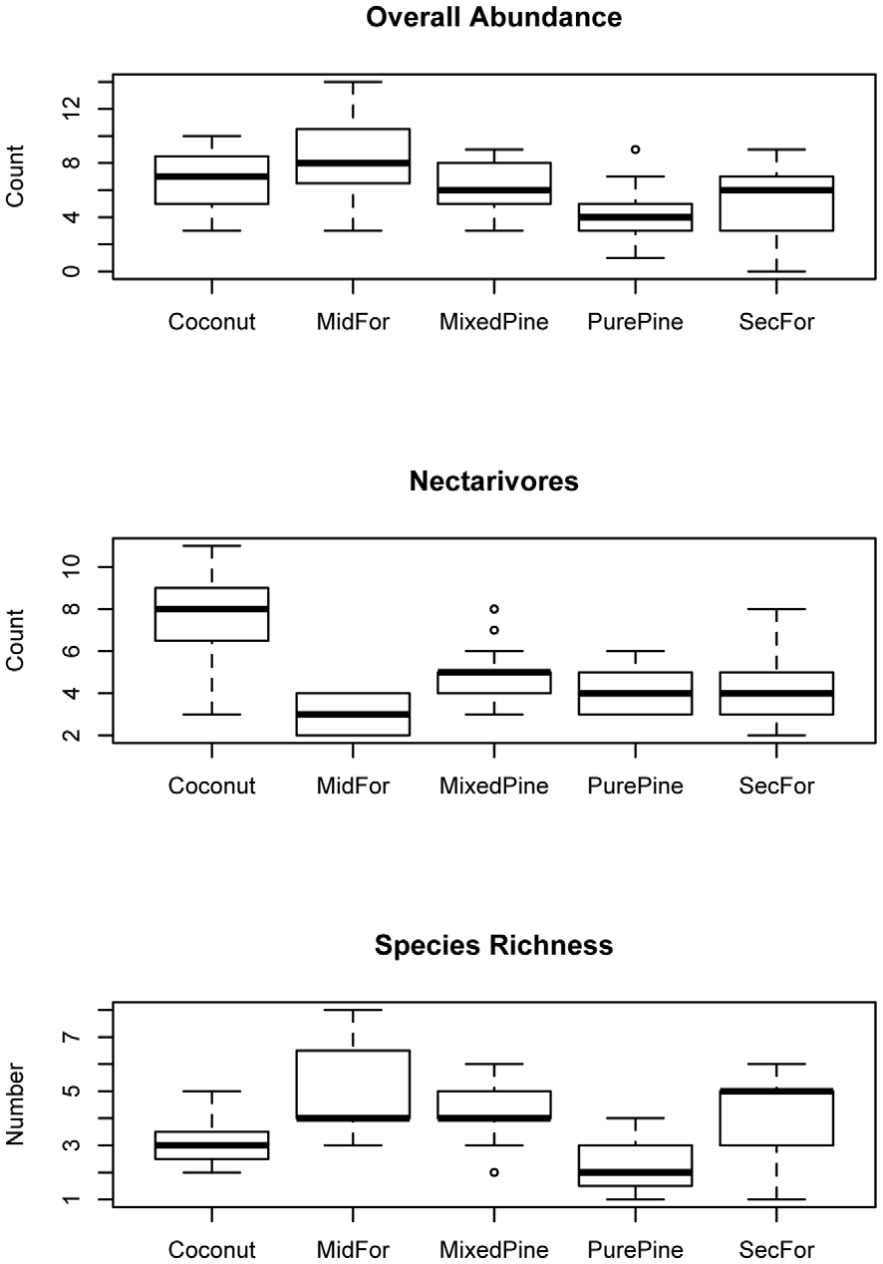
Box plots showing frequency distribution of abundance and richness of landbird communities in habitats on Lakeba. Frequency distribution of overall abundance, abundance of nectarivores (point counts), and overall species richness (number of species per point) per habitat (wet and dry season surveys lumped). Boxplots show the median (heavy line), quartiles (box) and 5^th^/95^th^ percentiles (whiskers). Habitat categories are: Coconut  =  Coastal Coconut Plantation; MidFor  =  Mid-successional Forest; MixedPine  =  Mixed Pine Woodland; PurePine  =  Pure Pine Woodland; SecFor  =  Secondary Forest (see Table 1).

The relative abundance of landbirds on Lakeba (Table 5) was not significantly different between seasons (fixed effect; P = 0.652) but differed among habitat types (nested within seasons; P≪0.001). This was primarily because abundance was lower in Pure Pine Woodland than in Mid-successional Forest (Figure 6). Although seasonal differences in total abundance were not significant overall, the data suggest that in modified habitats (Coastal Coconut Plantation and Pure and Mixed Pine Woodland) we detected more species in the wet season than the dry season (Table 5).

**Table 5:** Relative abundance of forest birds during the wet (18 February-6 March 2000) and dry season (10-15 May 1999) on Lakeba (five habitats) and Aiwa Levu (single habitat, single season).

Species/Guild	Lakeba: Coastal Coconut Plantation		Lakeba: Secondary Forest {Gp 2}		Lakeba: Mid-successional Forest {Gp 3, 4}		Lakeba: Mixed Pine Woodland		Lakeba: Pure Pine Woodland		Aiwa Levu: Coastal Forest {Gp 1}
	Wet (8)	Dry (7)	Wet (12)	Dry (9)	Wet (4)	Dry (9)	Wet (10)	Dry (9)	Wet (8)	Dry (10)	Wet (15)
*Circus approximans* R	*	-	0.1	0.2		-	*	-	-	-	*
*Falco peregrinus* R	-	-	-	-	-	-	-	-	-	-	**
*Gallirallus philippensis* GO	-	-	-	-	-	-	0.1	-	-	-	0.1
*Porphyrio porphyrio* GO	-	-	-	-	-	-	0.1	-	-	-	-
*Columba vitiensis* C	*	-	*	*	*	-	0.2	*	0.1	0.1	0.1
*Gallicolumba stairi* C	-	-	-	-	-	0.1	-	-	-	-	0.4
*Ptilinopus perousii* C	-	-	-	-	-	0.6	-	-	-	-	0.1
*Ptilinopus porphyraceus* C	-	*	0.2	0.4	0.2	1.0	0.1	*	*	-	0.5
*Ducula pacifica* C	*	-	0.2	0.1	1.0	0.9	-	-	-	-	1.9
*Vini solitarius* N	0.5	-	-	-	-	-	-	-	-	-	-
*Tyto alba* R	-	-	-	-	-	0.1	-	-	-	-	0.1
*Collocalia spodiopygia* NPI	3.1	0.6	0.8	0.9	1.5	0.1	1.3	3.4	0.6	0.9	0.1
*Halcyon chloris* NPI	0.4	0.3	0.3	0.4	1.0	0.4	0.5	0.4	0.1	0.1	1.2
*Aplonis tabuensis* PO	0.4	0.9	0.9	1.0	1.2	1.0	1.1	0.9	0.6	0.5	1.7
*Lalage maculosa* PO	-	-	-	-	-	-	0.1	0.4	-	-	0.1
*Mayrornis lessoni* PI	0.1	-	1.1	0.7	2.5	3.3	0.4	1.1	-	-	2.3
*Clytorhynchus vitiensis* PI	-	-	-	-	-	-	-	-	-	-	0.9
*Myiagra vanikorensis* PI	-	0.3	0.8	1.4	2.0	2.1	0.8	1.0	0.1	0.1	1.5
*Myzomela jugularis* N	3.4	2.1	1.2	1.0	-	0.3	1.8	1.6	1.6	1.3	-
*Foulehaio carunculata* N	2.9	2.4	0.8	1.8	1.0	0.7	1.2	1.0	0.9	0.3	1.8
TOTAL											
Relative abundance	7.6	6.0	5.5	7.0	9.0	10.5	6.4	6.4	3.5	2.4	12.7
Species (without *)	7	6	10	10	8	12	12	8	7	6	15
Species (with *)	10	7	12	11	9	12	13	10	8	6	17
Columbid	*	*	0.4	0.5	1.2	2.6	0.3	*	0.1	0.1	3.0
Passerine Insectivore	0	0.3	1.8	2.1	1.8	5.4	1.0	2.1	0.1	0.1	4.7
Nectarivore	6.8	4.5	1.9	2.8	1.0	1.0	3.0	2.6	2.5	1.6	1.8

Vegetation groups (Gp) from Table 2 are given in brackets. Data are expressed as mean individuals seen/heard per point, rounded to the nearest 0.1. The number of point counts is given in parentheses. *Collocalia* is excluded from Total relative abundance but not Total species (see Methods). An asterisk (*) denotes species recorded during point-counts but only at distances >50 m. Updated from Steadman & Franklin (2000), Steadman (2006: Table 6-6). Guild categories: C, columbid frugivore/granivore; GO, ground-dwelling omnivore; N, nectarivore; NPI, non-passerine insectivore; PI, passerine insectivore; PO, passerine omnivore; R, raptor.

**Figure 7:**
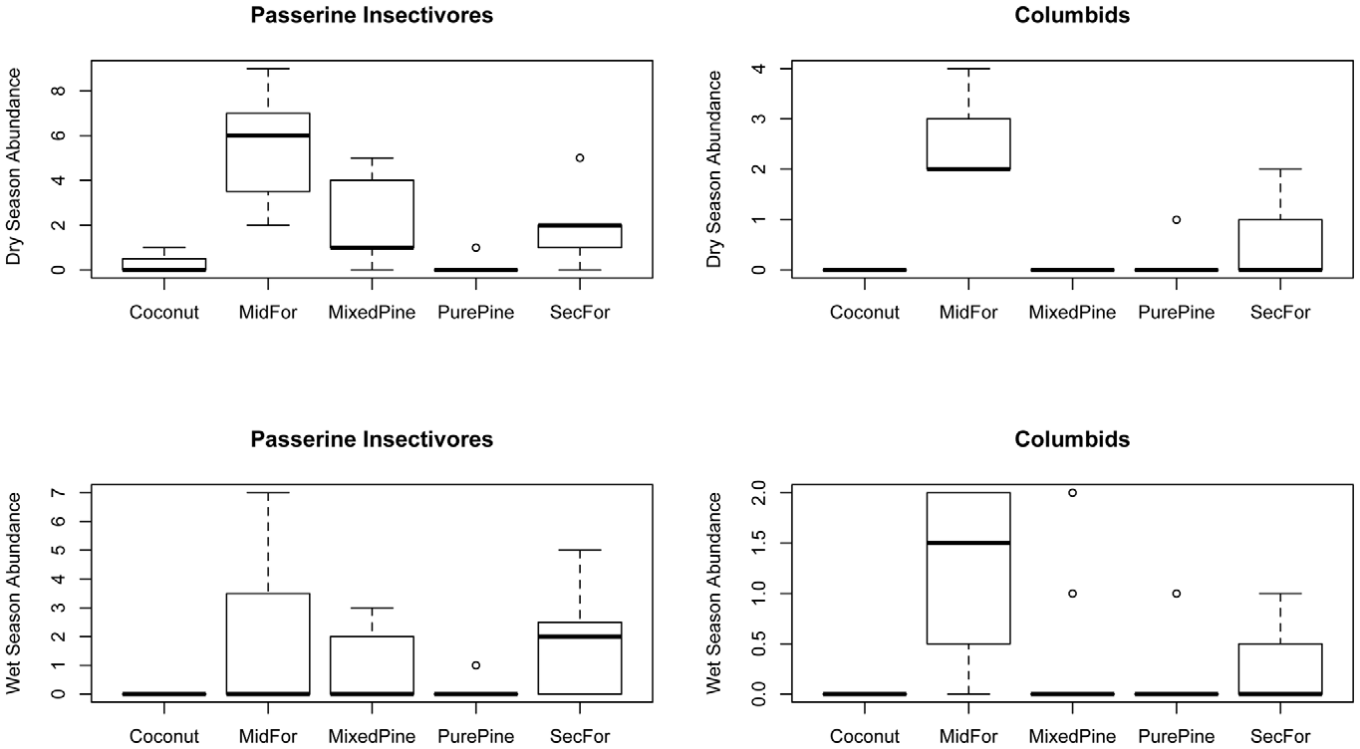
Box plots showing frequency distribution of abundance of guilds in habitats on Lakeba in the dry versus wet season. Passerine insectivores and columbid frugivores/granivores (point counts) in habitats for Lakeba in the dry season (May, top row) vs. wet season (Feb-Mar, bottom row). Habitat categories are defined and box plots explained in the caption of Figure 6 (see also Table 1). Note different scales on y-axes.

Differences in the abundance of columbid frugivores and granivores (pigeons and doves, i.e., *Ducula pacifica*, *Ptilinopus perousii*, *P*. *porphyraceus*, *Gallicolumba stairi*) were marginally significant among seasons (P = 0.083) and highly significantly among habitat types on Lakeba (P≪0.001), with more individuals recorded in the dry season, and more found in Mid-successional Forest than in any of the more disturbed habitats including Secondary Forest (Figure 7; Table 5). Similarly, the abundance of passerine insectivores (*Mayrornis lessoni*, *Myiagra vanikorensis*) was significantly greater in the dry season (P = 0.020), with more found in Mid-successional Forest than in either Pure Pine Woodland or Coastal Coconut Plantation (Figure 7). The relative abundance of passerine nectarivores (*Myzomela jugularis*, *Foulehaio carunculata*) was not significantly different between seasons (P = 0.53) but significant differences among habitats (P≪0.001) were the result of much higher abundance in Coastal Coconut Plantation than in any other habitat on Lakeba (Figure 6).

**Figure 8:**
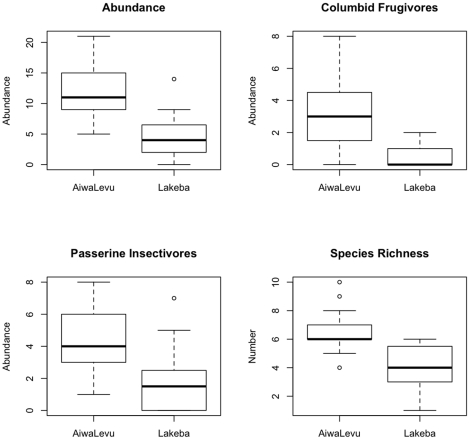
Box plots showing frequency distributions of forest birds of Lakeba versus Aiwa Levu in the wet season. Overall landbird abundance, abundance of columbid frugivores/granivores, abundance of passerine insectivores (point counts), and species richness (number of species) are shown (both islands sampled in Feb-Mar 2000). This comparison only includes points counts within native forest; 15 in Coastal Forest on Aiwa Levu, and 16 in Secondary Forest (12) + Mid-successional Forest (4) on Lakeba (Table 2). Note differences in scales on y-axes.

Although overall species richness was not significantly different between seasons on Lakeba (P = 0.66), average richness per point differed among habitats (P≪0.001) with a pattern that mirrored overall abundance (Figure 6), i.e., richness was greater in habitats with native trees than in purely anthropogenic habitats (Coastal Coconut Plantation, Pure Pine Woodland). This conclusion is unaffected by unequal sampling among habitats on Lakeba; total species richness estimated by rarefaction based on data pooled by seasons was lowest in Coastal Coconut Plantation (5.76±0.45) and Pure Pine Woodland (7), intermediate in Secondary Forest (8.50±0.61), and highest in Mixed Pine Woodland (9.88±1.08) and Mid-successional Forest (10.87±0.84) where richness was estimated for the lowest pooled abundance of 57 individuals detected (for Pure Pine Woodland). Average abundance (P<0.001) and species richness (P<0.001) were significantly greater on tiny Aiwa Levu than on much larger Lakeba when only forested habitats surveyed at the same time (wet season, Feb-Mar 2000) were compared (Table 5; Figure 8).

The overall relative abundance of birds among habitats on Nayau could not be analyzed statistically owing to limited sampling. Only three species were common in Mid-successional Forest on Nayau (the dove *Ptilinopus porphyraceus*, swiftlet *Collocalia spodiopygia*, and honeyeater *Foulehaio carunculata*). The latter was abundant in Coastal Coconut Plantation (75% of all individual birds detected on Nayau) because the coconut trees were flowering heavily in October. The overall scarcity of birds in Mid-successional Forest on Nayau stands in stark contrast to the situations in similar habitats on Lakeba and Aiwa Levu (Table 5 vs. Table 6).

**Table 6:** Relative abundance of birds on Nayau, Lau Group, Fiji.

Species/Guild	Nayau: Coastal Coconut Plantation (8)	Nayau: Secondary Forest {Group 2} (4)	Nayau: Mid-successional Forest {Groups 3 & 4} (4)
*Circus approximans* R	-	*	-
*Falco peregrinus* R	-	-	-
*Gallirallus philippensis* GO	0.2	0.5	-
*Porphyrio porphyrio* GO	0.1	-	-
*Columba vitiensis* C	0.1	0.2	0.1
*Gallicolumba stairi* C	-	-	-
*Ptilinopus porphyraceus* C	0.1	0.2	1.0
*Ptilinopus perousii* C	-	-	-
*Ducula pacifica* C	*	-	*
*Ducula latrans* C	-	-	0.2
*Vini solitarius* N	0.2	-	-
*Tyto alba* R	-	-	*
*Collocalia spodiopygia* NPI	0.6	0.8	1.0
*Halcyon chloris* NPI	0.6	*	0.1
*Aplonis tabuensis* PO	0.1	0.8	-
*Lalage maculosa* PO	-	-	-
*Mayrornis lessoni* PI	-	-	*
*Clytorhynchus vitiensis* PI	-	-	-
*Myiagra vanikorensis* PI	-	-	-
*Myzomela jugularis* N	-	1.5	-
*Foulehaio carunculata* N	4.9	2.2	0.9
			
TOTAL			
Relative abundance	6.5	5.5	2.4
Species (without *, **)	9	7	6
Species (with *, **)	10	9	9
Columbid abundance	0.2	0.5	1.4
Nectarivore abundance	4.9	3.8	0.9

Late dry season (23-24 October 2001).

Data are expressed as mean individuals seen/heard per point, rounded to the nearest 0.1. The number of point counts is given in parentheses for each time interval in each habitat type. *Collocalia* is excluded from Total relative abundance but not Total species. An asterisk (*) denotes species recorded during point-counts but only at distances >50 m;

**, occurs in this forest type but not recorded during point-counts. See Table 5 caption for guild categories.

## Discussion

### Forest Communities

The composition of forests surveyed on Lakeba and Nayau varied somewhat with topographic gradients, but much more with degrees of human disturbance, i.e., stages of secondary succession after clearing for agriculture or settlement, or other less intensive forms of forest disturbance, such as selective logging. Agricultural people settled these islands nearly 3000 years ago, and inland fortified sites were occupied for several periods as recently as 200 years ago [38], [40]. Thus forests were cleared even in upland rocky areas that are not agriculturally productive, and that now support relatively mature forest, such as at Vagadra on Lakeba [38]. Long-term cultivation in tropical forest sites affects community composition [62], [63]. With a multi-millenial agricultural history, forests in West Polynesia (WP) that retain old-growth characteristics occur in patches only in the most remote, inaccessible sites [23], [30], [32].

All of the 19 plots surveyed on Lakeba and Nayau fall within the Limestone Forest community (Table 1). We differentiated three forest types by the dominance of either *Alphitonia zizyphoides* (Secondary Forest; Group 2), *Gyrocarpus americanus* and *Dysoxylum* spp. (Mid-successional Forest; Group 3), or *Ficus prolixa* (Mid-successional Forest; Group 4). *Alphitonia zizyphoides* is an important early-successional tree in the region [61], [64], [65]. The widespread occurrence of *Cryptocarya hornei*, in contrast with the low frequency of *Macaranga* spp., suggests that Group 2 Secondary Forest sites have been regenerating for at least several decades. Species of *Cryptocarya* are typically shade tolerant [65], [66], [67] and do not establish in WP forests immediately following large scale disturbance; species of *Macaranga* are short lived, fast growing pioneers, and dominate only in the first decade or two after disturbance [61], [66], [68], [69], [70]. Lacking *Alphitonia*, *Cryptocarya*, and *Macaranga*, the two Group 4 plots represent the most mature forest sampled on Lakeba. Group 3 Mid-successional Forest shares its strong dominance by deciduous *Gyrocarpus americanus* with dry forest described for western Fiji [34].

While it is commonly recognized that forests on small islands can sustain endemic and endangered species of vertebrates [21], [71], the importance of such forests for endangered or even undescribed species of plants is less well appreciated. Even our limited sampling in Lau turned up two putative new species of *Syzygium* (Myrtaceae) [Table 2, and see 39]. Large forest trees including *Intsia bijuga*, *Burckella richii*, and *Callophyllum vitiense* are selectively cut on Nayau for house and boat building, so the forest there continues to lose its old growth characteristics.

### Inter-island Comparisons of Bird Communities in Lau

The relative abundance and species richness of birds on Lakeba are greatest in Mid-successional Forest, the habitat least affected by people. This is mostly due to increased numbers of columbids and passerine insectivores. By contrast, the relative abundance of passerine nectarivores is greatest in disturbed habitats with many coconut trees [cf. [72]]. In the two habitats most affected by people (Coastal Coconut Plantation, Pure Pine Woodland), we detected more individuals and species in the wet season than the dry season. In the Mid-successional Forest, by contrast, more individuals and species were detected in the dry season, in part because of increased vocalization by columbids, as has been reported in Samoa [33]. This may be only a seasonal difference in detectability rather than an actual population increase of columbids during the dry season.

Habitat quality influenced the landbird communities more than island area. Both richness and abundance of birds in forest were greater on very small Aiwa Levu than on Lakeba. This is in spite of the fact that Aiwa Levu's forest is more coastal in composition than Lakeba's (Table 2), and typically WP landbird abundance and diversity is lower in coastal forest with more littoral characteristics than in inland lowland forest [35]. Lakeba is mostly deforested and has 2500 people as well as chickens (*Gallus gallus*), Pacific rats (*Rattus exulans*), black rats (*R. rattus*), cats (*Felis catus*), dogs (*Canis familiaris*), horses (*Equus caballus*), pigs (*Sus scrofa*), and cows (*Bos taurus*). Aiwa Levu is uninhabited, 100% forested, and lacks non-native mammals except Pacific rats and goats (*Capra hircus*). To what extent the differences in non-native vertebrates affects the abundance and diversity of landbirds is not known. Columbids and passerine insectivores are more abundant per unit area on Aiwa Levu than on Lakeba (Table 5; Figure 8). Two species found on Aiwa Levu (*Falco peregrinus*, *Clytorhynchus vitiensis*) seem to be extirpated on Lakeba; *Myzomela jugularis* is the only species found in native forest on Lakeba that we did not detect on Aiwa Levu.

Nayau is mostly deforested and has 430 people as well as chickens, rats (species uncertain), cats, dogs, horses, pigs, and cows. From our brief visit in October 2001, the most distinctive aspects of Nayau's landbird fauna are the presence of the pigeon *Ducula latrans* (absent on Lakeba and Aiwa Levu) and an apparent island-wide absence or rarity of the passerines *Lalage maculosa*, *Mayrornis lessoni*, *Clytorhynchus vitiensis*, and *Myiagra vanikorensis*, which inhabit the other islands (except that we did not detect *C. vitiensis* on Lakeba).

Many bird populations on Pacific islands have been extirpated as a result of prehistoric human impacts [1], [73]. Because contemporary bird communities are also influenced by human impacts, which affect some islands more than others, we would expect prehistoric avifaunas to have been more similar in composition among the Lau islands than they are now [e.g., [1]]. Some data on prehistoric bird bones are available from our study islands although these data are compromised by large inter-island differences in the number of identified fossils and the ages of the sites [40]. Nevertheless, prehistoric bones increase the landbird fauna from 21 to 27 species on Lakeba, 18 to 20 species on Nayau, and 18 to 26 species on Aiwa Levu [1: Tables 6–5, 6–8]. On no single island in Lau is the fossil record nearly as thorough as that from the seven Tongan islands, ranging in size from 2–260 km^2^, where the prehistoric number of bird species varied little with island area before human impact [shown by rarefaction–based species–area calculations; [36]]. Therefore, it is not yet possible to say anything conclusive about the relationship between bird species richness and island area in central Lau, especially given that only on these three islands have modern or prehistoric avaifuanas been sampled. We note, however, that prehistoric species richness values are nearly identical on relatively large Lakeba and small Aiwa Levu.

### Conclusions

We identified variation in the composition of forest in Lau that corresponds to a gradient of habitat quality and degree of human disturbance. We discovered that the level of forest maturity is more important than island area in shaping the forest landbird communities of Lau. This effect is especially strong for pigeons and doves (which are important seed dispersers in Oceania [74], [75]) and passerine insectivores (the foraging ecology of which is not well known). Species richness and relative abundance of birds in the most mature forest types were higher on very small Aiwa Levu than on nearby Lakeba, which is more than an order of magnitude larger than Aiwa Levu and has ca. five times as much native forest cover today, but has experienced several millennia of human occupation resulting in extensive forest clearing and fragmentation. This supports the notion that conservation of vertebrates on tropical oceanic islands relies not only on identification of critical habitat for extant and translocated populations [12], [34], but also on protection from contemporary threats including habitat loss and disturbance and introduced predators [14], [76], [77].

While it has been recognized that even small islands can sustain endemic and endangered species of vertebrates, such forests are also important sites for endangered or even undiscovered species of plants, as further exploration of the remote islands of Lau will undoubtedly confirm. Such a plea for new field work may be time-limited; although the forests here still consist primarily of native species, without the problem of invasive trees as in parts of Samoa [28] and large islands of western Fiji [78], the appearance of invasive non-native trees could occur at any time.

## Supporting Information

Figure S1 **Species accumulation curve.** Estimated rate of accumulation of new species for 23 vegetation plots on Lakeba, Nayau and Aiwa Levu islands, Lau Group, Fiji. Note that the number of species accumulates rapidly from 1 to ∼10 plots and then begins to level off (the rate of accumulation slows). The estimated number of species in the sampled forests is 112-127 using first and second-order jackknife estimates (see text).
(DOC)

Table S1 Basal area by species (square m/ha) and environmental and stand variables for 23 forest plots in Lau, each 500 m^2^ in area.
(DOC)
