# Supplementary material for: Forest Plant and Bird Communities in the Lau Group, Fiji
Source: PLoS One. 2010 Dec 29;5(12):e15685. doi: 10.1371/journal.pone.0015685 (PMC3012085; doi:10.1371/journal.pone.0015685)
Supplement: Figure S1 — Species accumulation curve. Estimated rate of accumulation of new species for 23 vegetation plots on Lakeba, Nayau and Aiwa Levu islands, Lau Group, Fiji. Note that the number of species accumulates rapidly from 1 to ∼10 plots and then begins to level off (the rate of accumulation slows). The estimated number of species in the sampled forests is 112-127 using first and second-order jackknife estimates (see text). (DOC) [file pone.0015685.s001.doc]

Supplementary Material. Franklin, J., and Steadman, D. W.

**Figure S1** Species accumulation curve showing estimated rate of accumulation of new species for 23 vegetation plots on Lakeba, Nayau and Aiwa Levu islands, Lau Group, Fiji. Note that the number of species accumulates rapidly from 1 to ~10 plots and then begins to level off (the rate of accumulation slows). The estimated number of species in the sampled forests is 112-127 using first and second-order jackknife estimates (see text).
