# Supplementary material for: Forest Plant and Bird Communities in the Lau Group, Fiji
Source: PLoS One. 2010 Dec 29;5(12):e15685. doi: 10.1371/journal.pone.0015685 (PMC3012085; doi:10.1371/journal.pone.0015685)
Supplement: Table S1 — Basal area by species (square m/ha) and environmental and stand variables for 23 forest plots in Lau, each 500 m2 in area. (DOC) [file pone.0015685.s002.doc]

**Table S1** For 23 500 m-squared forest plots in Lau, basal area by species (square m/ha) and environmental and stand variables

| Tree Species (Authorship) or Environmental Variable | Family (Species) or Units (Environmental) | P1 aiwa | P2 aiwa | P3 aiwa | P4 aiwa | P5 vaga | P6 tara | P7 tara | P8 tara | P9 tara | P10 vaga | P11 vaga | P12 vaga | P13 vaga | P14 tara |
| --- | --- | --- | --- | --- | --- | --- | --- | --- | --- | --- | --- | --- | --- | --- | --- |
| *Acalypha insulana* Müll.Arg. | EUPHORBIACEAE | 0.00 | 0.00 | 0.00 | 0.00 | 0.00 | 0.00 | 0.00 | 0.00 | 0.00 | 0.00 | 0.00 | 0.00 | 0.00 | 0.00 |
| *Adenanthera pavonina* L | MIMOSACEAE | 0.00 | 0.00 | 0.00 | 0.00 | 0.00 | 3.20 | 0.36 | 0.00 | 0.00 | 0.00 | 0.56 | 0.00 | 0.00 | 0.00 |
| *Aglaia saltatorum* A.C.Sm. | MELIACEAE | 0.99 | 2.05 | 0.00 | 1.98 | 0.00 | 0.00 | 0.00 | 0.00 | 0.00 | 0.27 | 0.04 | 0.00 | 0.00 | 0.58 |
| *Alangium vitiense*  (A.Gray) Baill. ex Harms | ALANGIACEAE | 0.00 | 0.00 | 0.00 | 0.00 | 0.00 | 0.00 | 0.00 | 0.00 | 0.00 | 0.18 | 0.00 | 5.65 | 0.00 | 0.00 |
| *Allophylus timorensis* (DC.) Blume | SAPINDACEAE | 0.00 | 0.00 | 0.00 | 0.00 | 0.00 | 0.00 | 0.00 | 0.00 | 0.00 | 0.00 | 0.00 | 0.00 | 0.00 | 0.00 |
| *Alphitonia zizyphoides* (Spreng.) A.Gray | RHAMNACEAE | 0.00 | 0.00 | 0.00 | 0.00 | 0.00 | 10.07 | 0.00 | 5.12 | 0.00 | 0.00 | 0.00 | 0.00 | 0.00 | 0.00 |
| *Antirhea inconspicua* (Seem.) Christoph. | RUBIACEAE | 0.00 | 0.00 | 0.00 | 0.00 | 0.00 | 0.05 | 0.00 | 0.00 | 2.23 | 0.00 | 0.00 | 0.00 | 0.00 | 0.00 |
| *Arytera brackenridgei* (A.Gray) Radlk. | SAPINDACEAE | 0.00 | 0.00 | 0.00 | 0.00 | 3.04 | 0.00 | 0.00 | 0.00 | 0.00 | 0.00 | 0.21 | 0.00 | 0.00 | 0.00 |
| *Barringtonia edulis* Seem. | LECYTHIDACEAE | 0.00 | 0.00 | 0.00 | 0.00 | 0.00 | 0.00 | 0.00 | 0.00 | 0.00 | 0.07 | 0.00 | 0.00 | 0.30 | 9.34 |
| *Buchanania vitiensis* Engl. | ANACARDIACEAE | 0.00 | 0.00 | 0.00 | 0.00 | 0.00 | 0.47 | 0.00 | 0.00 | 9.54 | 0.09 | 0.00 | 0.00 | 0.00 | 0.00 |
| *Burckella richii* (A.Gray) H.J.Lam | SAPOTACEAE | 0.00 | 0.00 | 0.00 | 0.00 | 0.00 | 0.00 | 1.56 | 0.00 | 0.00 | 0.00 | 0.00 | 0.00 | 0.00 | 0.00 |
| *Calophyllum neo-ebudicum* Guillaumin | CLUSIACEAE | 0.00 | 0.00 | 0.00 | 0.00 | 0.00 | 0.00 | 0.00 | 0.00 | 0.00 | 0.00 | 0.00 | 0.00 | 0.00 | 0.00 |
| *Canarium harveyi* Seem. | BURSERACEAE | 0.00 | 0.00 | 0.00 | 0.00 | 0.00 | 0.00 | 0.00 | 0.00 | 0.00 | 0.00 | 0.00 | 0.00 | 0.00 | 0.00 |
| *Cerbera manghas* L. | APOCYNACEAE | 0.00 | 0.00 | 0.00 | 0.00 | 0.00 | 0.00 | 0.00 | 0.00 | 1.72 | 0.00 | 0.00 | 0.00 | 0.00 | 0.00 |
| *Claoxylon fallax* Müll.Arg. | EUPHORBIACEAE | 0.00 | 0.00 | 0.00 | 0.00 | 0.00 | 0.00 | 0.00 | 0.00 | 0.00 | 0.00 | 0.00 | 0.00 | 0.00 | 0.00 |
| *Cocos nucifera* L. | ARECACEAE | 0.00 | 0.00 | 0.00 | 0.00 | 0.00 | 1.49 | 0.00 | 1.38 | 1.34 | 0.00 | 0.00 | 0.00 | 0.00 | 2.89 |
| *Cordyline fruticosa* (L.) A.Chev. | AGAVACEAE | 0.00 | 0.00 | 0.00 | 0.00 | 0.00 | 0.00 | 0.00 | 0.00 | 0.14 | 0.00 | 0.00 | 0.00 | 0.00 | 0.00 |
| *Cryptocarya hornei* Gillespie | LAURACEAE | 0.27 | 0.00 | 0.00 | 0.27 | 0.00 | 0.00 | 3.19 | 0.04 | 0.00 | 2.53 | 0.31 | 2.60 | 0.00 | 0.00 |
| *Cyclophyllum barbatum* (G.Forst.) A.C.Sm. & S.P.Darwin | RUBIACEAE | 0.00 | 0.00 | 0.00 | 0.00 | 0.00 | 0.00 | 0.00 | 0.00 | 0.00 | 0.00 | 0.00 | 0.00 | 0.00 | 0.00 |
| *Dendrocnide harveyi* (Seem.) Chew | URTICACEAE | 0.00 | 0.00 | 0.00 | 0.00 | 0.10 | 0.00 | 0.00 | 0.00 | 0.08 | 0.00 | 0.00 | 0.00 | 0.00 | 0.00 |
| *Diospyros elliptica* (J.R.Forst & G.Forst.) P.S.Green | EBENACEAE | 0.00 | 0.08 | 0.51 | 0.06 | 0.00 | 0.00 | 0.00 | 0.00 | 0.00 | 0.00 | 0.00 | 0.00 | 0.00 | 0.00 |
| *Diospyros samoensis* A.Gray | EBENACEAE | 1.34 | 0.95 | 2.46 | 0.00 | 0.00 | 0.00 | 0.36 | 0.00 | 0.86 | 0.09 | 0.00 | 0.00 | 0.00 | 0.00 |
| *Diospyros vitiensis* var. *longisepala* (Gillespie) A.C.Sm. | EBENACEAE | 0.00 | 0.00 | 0.00 | 0.00 | 0.00 | 0.00 | 0.00 | 0.00 | 0.00 | 0.00 | 0.00 | 0.00 | 0.00 | 0.00 |
| *Dysoxylum richii* (A.Gray) C.DC. | MELIACEAE | 6.06 | 7.99 | 0.00 | 4.98 | 3.66 | 1.74 | 0.00 | 1.99 | 0.91 | 39.59 | 6.69 | 5.87 | 0.00 | 7.31 |
| *Dysoxylum tenuiflorum* A.C.Sm. | MELIACEAE | 0.00 | 0.00 | 0.00 | 0.00 | 13.49 | 0.00 | 0.00 | 0.00 | 0.00 | 0.00 | 15.04 | 14.32 | 5.36 | 0.00 |
| *Elattostachys falcata* (A.Gray) Radlk. | SAPINDACEAE | 0.00 | 0.00 | 0.00 | 0.00 | 0.00 | 0.00 | 0.39 | 0.00 | 0.00 | 0.00 | 3.44 | 0.00 | 0.00 | 0.00 |
| *Erythrospermum acuminatissimum* (A.Gray) A.C.Sm. | FLACOURTIACEAE | 0.00 | 0.00 | 0.00 | 0.00 | 0.00 | 0.00 | 0.00 | 0.00 | 0.00 | 0.00 | 0.00 | 0.00 | 0.00 | 0.00 |
| *Eugenia reinwardtiana* (Blume) DC. | MYRTACEAE | 0.00 | 0.10 | 0.00 | 0.06 | 0.00 | 0.00 | 0.14 | 0.00 | 0.00 | 0.00 | 0.00 | 0.00 | 0.00 | 0.00 |
| *Ficus masonii* Horne ex Baker | MORACEAE | 0.00 | 0.00 | 0.00 | 0.00 | 0.00 | 0.00 | 0.00 | 0.00 | 0.00 | 0.00 | 0.00 | 0.00 | 0.52 | 0.00 |
| *Ficus obliqua* G.Forst. | MORACEAE | 0.00 | 0.00 | 7.70 | 0.00 | 0.00 | 0.00 | 0.00 | 0.00 | 0.00 | 0.00 | 0.00 | 0.00 | 0.00 | 0.00 |
| *Ficus prolixa* G.Forst. | MORACEAE | 0.00 | 0.00 | 0.00 | 0.00 | 392.70 | 0.00 | 0.00 | 0.00 | 62.83 | 0.00 | 0.00 | 0.00 | 0.00 | 0.00 |
| *Ficus scabra* G.Forst. | MORACEAE | 0.00 | 0.00 | 0.00 | 0.76 | 1.27 | 0.38 | 1.64 | 1.03 | 0.00 | 0.41 | 0.00 | 1.28 | 0.00 | 1.43 |
| *Flacourtia subintegra* A.C.Sm. | FLACOURTIACEAE | 0.00 | 0.00 | 0.00 | 0.00 | 0.00 | 0.00 | 0.00 | 0.00 | 0.00 | 0.00 | 0.00 | 0.00 | 0.00 | 0.00 |
| *Garcinia* *sessilis* (G.Forst.) Seem. | CLUSIACEAE | 0.00 | 0.00 | 0.00 | 0.00 | 0.00 | 0.00 | 0.00 | 0.00 | 0.00 | 0.00 | 0.00 | 0.00 | 0.00 | 0.00 |
| *Garuga floribunda* Decne. | BURSERACEAE | 0.00 | 0.00 | 0.00 | 0.00 | 0.00 | 0.00 | 0.00 | 0.00 | 0.00 | 0.00 | 0.00 | 0.00 | 0.00 | 0.00 |
| *Geniostoma rupestre* J.R.Forst. & G.Forst. | LOGANIACEAE | 0.00 | 0.00 | 0.00 | 0.00 | 0.00 | 0.00 | 0.00 | 0.00 | 0.00 | 0.00 | 0.00 | 0.00 | 0.00 | 0.00 |
| *Glochidion concolor* Müll.Arg. | EUPHORBIACEAE | 0.00 | 0.00 | 0.00 | 0.00 | 0.00 | 0.00 | 0.00 | 0.00 | 0.00 | 0.00 | 0.00 | 0.00 | 0.00 | 0.00 |
| *Guettarda speciosa* L. | RUBIACEAE | 0.98 | 0.00 | 1.41 | 0.00 | 0.00 | 0.00 | 0.00 | 0.00 | 0.00 | 0.00 | 0.00 | 0.00 | 0.00 | 0.00 |
| *Guioa rhoifolia* (A.Gray) Radlk. | SAPINDACEAE | 0.00 | 0.00 | 0.00 | 0.00 | 0.00 | 0.00 | 0.69 | 0.00 | 0.35 | 0.00 | 0.00 | 0.00 | 0.00 | 0.00 |
| *Gyrocarpus americanus* Jacq. | HERNANDIACEAE | 0.00 | 0.00 | 0.00 | 0.00 | 0.83 | 0.00 | 17.90 | 0.00 | 0.00 | 16.77 | 3.04 | 0.00 | 19.01 | 37.48 |
| *Harpullia arborea* (Blanco) Radlk. | SAPINDACEAE | 1.26 | 0.00 | 0.00 | 6.05 | 0.00 | 0.00 | 0.39 | 0.00 | 0.00 | 0.00 | 0.00 | 0.00 | 0.00 | 0.00 |
| *Heritiera ornithocephala* Kosterm. | STERCULIACEAE | 0.00 | 0.00 | 0.00 | 0.00 | 0.00 | 0.00 | 0.00 | 0.00 | 0.00 | 0.00 | 0.00 | 0.00 | 0.00 | 0.00 |
| *Hibiscus tiliaceus* L. | MALVACEAE | 0.00 | 0.00 | 0.00 | 0.00 | 0.00 | 0.48 | 2.39 | 4.17 | 1.07 | 0.00 | 0.00 | 0.00 | 0.00 | 0.00 |
| *Homalium pallidum* A.C.Sm. | FLACOURTIACEAE | 0.00 | 0.00 | 0.00 | 0.00 | 0.00 | 0.00 | 0.00 | 0.00 | 0.00 | 0.00 | 0.00 | 0.00 | 0.00 | 0.00 |
| *Intsia bijuga* (Colebr.) Kuntze | CAESALPINIACEAE | 0.00 | 0.00 | 0.00 | 0.00 | 0.00 | 0.00 | 0.00 | 0.00 | 0.00 | 0.00 | 0.00 | 0.00 | 0.00 | 0.00 |
| *Macaranga harveyana* (Müll.Arg.) Müll.Arg. | EUPHORBIACEAE | 0.00 | 0.00 | 0.00 | 0.00 | 0.00 | 0.00 | 0.00 | 3.58 | 0.00 | 0.00 | 0.00 | 0.00 | 0.00 | 1.38 |
| *Macaranga seemannii* (Müll.Arg.) Müll.Arg. | EUPHORBIACEAE | 0.00 | 0.00 | 0.00 | 0.00 | 0.00 | 0.00 | 0.00 | 0.00 | 0.00 | 0.00 | 0.00 | 0.00 | 0.00 | 0.00 |
| *Mangifera indica* L. | ANACARDIACEAE | 0.00 | 0.00 | 0.00 | 0.00 | 0.00 | 10.00 | 0.00 | 0.00 | 0.00 | 0.00 | 0.00 | 0.00 | 0.00 | 0.00 |
| *Manilkara vitiensis* (H.J.Lam & v.Olden) Meeuse | SAPOTACEAE | 0.00 | 0.00 | 0.00 | 0.00 | 0.00 | 0.00 | 0.00 | 0.00 | 0.00 | 0.00 | 0.00 | 0.00 | 0.00 | 0.00 |
| *Maniltoa floribunda* A.C.Sm. | CAESALPINIACEAE | 0.00 | 2.73 | 0.79 | 6.57 | 0.00 | 0.00 | 0.00 | 0.00 | 0.00 | 0.00 | 0.00 | 0.54 | 0.00 | 0.00 |
| *Melicope cucullata* (Gillespie) A.C.Sm. | RUTACEAE | 0.00 | 0.00 | 0.00 | 0.00 | 0.00 | 56.93 | 0.00 | 2.56 | 0.30 | 0.00 | 0.00 | 0.00 | 0.00 | 0.00 |
| *Melochia vitiensis* A.Gray | STERCULIACEAE | 0.00 | 0.00 | 0.00 | 0.00 | 0.00 | 0.00 | 0.00 | 1.14 | 0.00 | 0.00 | 0.00 | 0.00 | 0.00 | 0.00 |
| *Millettia pinnata* (L.) Panigrahi (syn. *Pongamia pinnata* (L.) Pierre) | FABACEAE | 0.00 | 0.00 | 5.44 | 0.00 | 0.00 | 0.00 | 1.21 | 0.00 | 0.00 | 0.89 | 0.00 | 0.00 | 0.00 | 0.00 |
| *Myristica* *gillespieana* A.C.Sm. | MYRISTICACEAE | 0.00 | 0.00 | 0.00 | 0.00 | 0.00 | 0.00 | 0.00 | 0.00 | 0.00 | 0.00 | 0.00 | 0.00 | 5.62 | 0.00 |
| *Neisosperma oppositifolia* (Lam.) Fosberg & Sachet | APOCYNACEAE | 0.06 | 0.00 | 0.00 | 0.00 | 0.00 | 0.00 | 0.00 | 0.71 | 0.00 | 0.00 | 0.00 | 0.00 | 0.00 | 0.00 |
| *Neonauclea forsteri* (Seem. ex Havil.) Merr. | RUBIACEAE | 0.00 | 0.00 | 0.00 | 0.00 | 0.00 | 0.00 | 0.00 | 0.00 | 0.00 | 0.00 | 0.00 | 0.00 | 0.00 | 0.00 |
| *Pandanus tectorius* Parkinson | PANDANACEAE | 0.56 | 0.00 | 0.00 | 0.00 | 0.00 | 0.00 | 0.00 | 3.40 | 0.00 | 0.00 | 0.00 | 0.00 | 0.00 | 0.00 |
| *Phaleria pubiflora* (A.Gray) Gilg | THYMELAEACEAE | 0.00 | 0.00 | 0.00 | 0.00 | 0.00 | 0.00 | 0.00 | 0.00 | 0.37 | 0.00 | 0.00 | 0.00 | 0.00 | 0.00 |
| *Pisonia grandis* R.Br. | NYCTAGINACEAE | 7.30 | 29.29 | 0.00 | 8.37 | 0.00 | 0.00 | 0.00 | 0.00 | 0.00 | 0.00 | 0.00 | 0.00 | 0.00 | 0.00 |
| *Pittosporum arborescens* Rich ex A.Gray | PITTOSPORACEAE | 0.00 | 0.00 | 0.00 | 0.00 | 0.00 | 0.93 | 0.00 | 0.00 | 0.15 | 0.05 | 0.00 | 0.00 | 0.00 | 0.00 |
| *Pleiogynium timoriense* (DC.) Leenh. | ANACARDIACEAE | 0.00 | 0.00 | 0.00 | 0.00 | 0.00 | 0.00 | 0.00 | 0.00 | 0.00 | 0.00 | 0.00 | 0.00 | 0.00 | 0.00 |
| *Polyalthia laddiana* A.C.Sm. | ANNONACEAE | 0.06 | 0.00 | 0.06 | 0.00 | 0.00 | 0.00 | 1.82 | 0.00 | 0.33 | 2.09 | 0.00 | 0.00 | 0.00 | 0.04 |
| *Polyalthia vitiensis* Seem. | ANNONACEAE | 0.00 | 0.00 | 0.00 | 0.00 | 0.00 | 0.00 | 0.00 | 0.00 | 0.00 | 0.00 | 0.00 | 0.00 | 0.00 | 0.00 |
| *Polyscias multijuga* (A.Gray) Harms | ARALIACEAE | 0.00 | 0.00 | 0.00 | 0.00 | 0.00 | 0.00 | 0.22 | 0.00 | 0.90 | 0.05 | 0.04 | 0.00 | 0.00 | 0.64 |
| *Pometia pinnata* J.R.Forst. & G.Forst. | SAPINDACEAE | 0.00 | 0.00 | 0.00 | 0.00 | 0.00 | 0.00 | 0.00 | 0.00 | 0.00 | 0.00 | 0.00 | 0.00 | 0.00 | 0.00 |
| *Pouteria grayana* (H.St.John) Fosberg (syn. *Planchonella grayana* H.St.John) | SAPOTACEAE | 0.31 | 0.00 | 16.14 | 0.00 | 0.00 | 0.00 | 13.46 | 0.00 | 0.05 | 0.08 | 0.00 | 0.00 | 0.00 | 0.20 |
| *Premna serratifolia* L. | VERBENACEAE | 0.00 | 0.00 | 0.00 | 0.00 | 0.00 | 0.00 | 0.00 | 0.00 | 0.00 | 0.00 | 0.00 | 0.00 | 0.00 | 0.00 |
| *Serianthes melanesica* Fosberg | MIMOSACEAE | 0.00 | 0.00 | 0.00 | 0.00 | 0.00 | 0.00 | 0.00 | 0.00 | 0.00 | 0.00 | 0.00 | 0.00 | 0.00 | 0.00 |
| *Syzygium* aff. g*racilipes (undescribed)* | MYRTACEAE | 0.00 | 0.00 | 0.00 | 0.00 | 0.08 | 0.00 | 0.00 | 0.00 | 0.00 | 0.00 | 0.00 | 0.00 | 0.00 | 0.00 |
| *Syzygium quadrangulatum* (A.Gray) Merr. & L.M.Perry | MYRTACEAE | 0.00 | 0.00 | 0.00 | 0.00 | 0.00 | 0.00 | 0.00 | 0.00 | 0.00 | 0.00 | 0.00 | 0.00 | 0.00 | 0.00 |
| *Syzygium richii* (A.Gray) Merr. & L.M.Perry | MYRTACEAE | 0.00 | 0.00 | 0.00 | 0.00 | 0.00 | 0.00 | 0.00 | 0.00 | 0.00 | 0.00 | 0.00 | 0.00 | 0.00 | 0.00 |
| *Syzygium seemannii* (A.Gray) Biffin & Craven | MYRTACEAE | 0.00 | 0.00 | 0.00 | 0.00 | 0.00 | 0.00 | 0.00 | 0.00 | 0.00 | 0.00 | 0.00 | 0.00 | 0.00 | 0.00 |
| *Syzgium* sp. Nova (undescribed) | MYRTACEAE | 0.00 | 0.00 | 0.00 | 0.00 | 0.00 | 0.00 | 0.00 | 0.00 | 3.15 | 0.00 | 0.00 | 0.00 | 0.00 | 0.00 |
| *Tabernaemontana pandacaqui* Lam. (syn. *Ervatamia obtusiuscula* Markgr.) | APOCYNACEAE | 1.87 | 2.21 | 1.49 | 1.19 | 0.00 | 0.00 | 0.00 | 0.00 | 0.00 | 0.00 | 0.00 | 0.00 | 0.00 | 0.00 |
| *Terminalia catappa* L. | COMBRETACEAE | 0.00 | 0.00 | 0.00 | 0.00 | 0.00 | 0.00 | 3.31 | 0.00 | 0.00 | 0.00 | 0.00 | 0.00 | 0.00 | 0.00 |
| *Vavaea amicorum* Benth. | MELIACEAE | 0.00 | 0.00 | 0.13 | 0.00 | 0.00 | 0.00 | 0.61 | 0.00 | 1.55 | 0.26 | 0.00 | 0.00 | 0.00 | 0.00 |
| *Xylosma simulans* A.C.Sm. | FLACOURTIACEAE | 6.44 | 6.98 | 6.45 | 7.45 | 0.00 | 0.00 | 0.00 | 0.00 | 1.08 | 0.00 | 0.00 | 1.33 | 0.00 | 0.00 |
| Indeterminate Flacourtiaceae 41 | FLACOURTIACEAE | 0.00 | 0.00 | 0.00 | 0.00 | 0.00 | 0.00 | 0.00 | 0.00 | 0.00 | 0.00 | 0.00 | 0.00 | 0.00 | 0.00 |
| Indeterminate Lauraceae 20-16 | LAURACEAE | 0.00 | 0.00 | 0.00 | 0.00 | 0.00 | 0.00 | 0.00 | 0.00 | 0.00 | 0.00 | 0.00 | 0.00 | 0.00 | 0.00 |
| Indeterminate Lauraceae 21-5 | LAURACEAE | 0.00 | 0.00 | 0.00 | 0.00 | 0.00 | 0.00 | 0.00 | 0.00 | 0.00 | 0.00 | 0.00 | 0.00 | 0.00 | 0.00 |
| Indeterminate Lauraceae cf. Litsea sp. | LAURACEAE | 0.00 | 0.00 | 0.00 | 0.00 | 0.00 | 0.00 | 0.00 | 0.00 | 0.00 | 0.00 | 0.00 | 0.00 | 0.00 | 0.00 |
| Indeterminate Pisonia sp. | NYCTAGINACEAE | 0.00 | 0.00 | 0.00 | 0.00 | 0.00 | 0.00 | 0.00 | 0.00 | 0.00 | 0.00 | 0.00 | 0.00 | 0.00 | 0.00 |
| Indeterminate Syzygium sp. | MYRTACEAE | 0.00 | 0.00 | 0.00 | 0.00 | 0.00 | 0.00 | 0.00 | 0.00 | 0.00 | 0.00 | 0.00 | 0.00 | 0.14 | 0.00 |
| Indeterminate family 17-15 | unknown | 0.00 | 0.00 | 0.00 | 0.00 | 0.00 | 0.00 | 0.00 | 0.00 | 0.00 | 0.00 | 0.00 | 0.00 | 0.00 | 0.00 |
| Indeterminate family 18-3 | unknown | 0.00 | 0.00 | 0.00 | 0.00 | 0.00 | 0.00 | 0.00 | 0.00 | 0.00 | 0.00 | 0.00 | 0.00 | 0.00 | 0.00 |
| Forest Group | unitless, 1-4 (see text) | 1 | 1 | 1 | 1 | 4 | 2 | 3 | 2 | 4 | 3 | 3 | 3 | 3 | 3 |
| Slope | degrees | 0 | 0 | 10 | 0 | 15 | 0 | 0 | 10 | 0 | 25 | 3 | 8 | 0 | 2 |
| Aspect | degrees | 0.1 | 0.1 | 45 | 0.1 | 340 | 0.1 | 0.1 | 200 | 0.1 | 315 | 0.1 | 180 | 0.1 | 25 |
| Elevation | meters | 30 | 30 | 15 | 40 | 60 | 25 | 45 | 20 | 20 | 90 | 80 | 70 | 50 | 15 |
| Rockiness | percent cover | 0 | 0 | 20 | 3 | 25 | 0 | 40 | 5 | 0 | 50 | 5 | 10 | 0 | 90 |
| Canopy Height | meters | 12 | 12 | 15 | 12 | 15 | 15 | 13 | 9 | 14 | 22 | 22 | 12 | 25 | 12 |
| Canopy cover | percent cover | 80 | 80 | 85 | 80 | 95 | 70 | 90 | 65 | 85 | 90 | 80 | 80 | 90 | 80 |
| Total Basal Area | square meters per ha | 21.05 | 45.40 | 35.99 | 30.27 | 415.18 | 85.72 | 49.03 | 25.12 | 86.32 | 63.18 | 29.37 | 30.27 | 30.95 | 61.29 |

| Table 1, continued | | | | |  |  |  |  |  |  |
| --- | --- | --- | --- | --- | --- | --- | --- | --- | --- | --- |
| Tree Species (Authorship) or Environmental Variable | Family (Species) or Units (Environmental) | P15 tara | P16 vaga | P17 nayau | P18 nayau | P19 nayau | P20 nayau | P21 nayau | P22 nayau | P23 nayau |
| *Acalypha insulana* Müll.Arg. | EUPHORBIACEAE | 0.00 | 0.00 | 0.00 | 0.00 | 0.00 | 0.00 | 0.54 | 0.50 | 1.01 |
| *Adenanthera pavonina* L | MIMOSACEAE | 0.00 | 0.00 | 0.00 | 0.00 | 0.00 | 0.00 | 0.00 | 0.00 | 0.00 |
| *Aglaia saltatorum* A.C.Sm. | MELIACEAE | 0.17 | 0.00 | 0.00 | 0.00 | 0.00 | 0.00 | 0.00 | 0.00 | 0.00 |
| *Alangium vitiense*  (A.Gray) Baill. ex Harms | ALANGIACEAE | 0.00 | 0.00 | 0.20 | 0.06 | 2.85 | 0.17 | 1.01 | 0.43 | 0.82 |
| *Allophylus timorensis* (DC.) Blume | SAPINDACEAE | 0.00 | 0.00 | 0.00 | 0.00 | 0.15 | 0.00 | 0.00 | 0.00 | 0.00 |
| *Alphitonia zizyphoides* (Spreng.) A.Gray | RHAMNACEAE | 15.24 | 0.00 | 0.63 | 0.00 | 6.95 | 6.89 | 5.20 | 3.57 | 0.00 |
| *Antirhea inconspicua* (Seem.) Christoph. | RUBIACEAE | 0.10 | 1.23 | 0.00 | 0.00 | 0.00 | 0.00 | 0.00 | 0.06 | 0.00 |
| *Arytera brackenridgei* (A.Gray) Radlk. | SAPINDACEAE | 0.05 | 0.00 | 0.00 | 0.00 | 0.00 | 0.00 | 0.00 | 0.00 | 0.00 |
| *Barringtonia edulis* Seem. | LECYTHIDACEAE | 0.00 | 0.00 | 0.00 | 6.39 | 2.97 | 0.00 | 1.63 | 0.00 | 1.33 |
| *Buchanania vitiensis* Engl. | ANACARDIACEAE | 5.70 | 0.00 | 0.00 | 0.00 | 0.00 | 0.00 | 0.00 | 0.00 | 0.00 |
| *Burckella richii* (A.Gray) H.J.Lam | SAPOTACEAE | 0.00 | 0.00 | 0.00 | 5.28 | 0.34 | 5.82 | 0.00 | 0.00 | 2.49 |
| *Calophyllum neo-ebudicum* Guillaumin | CLUSIACEAE | 0.00 | 0.00 | 0.00 | 0.00 | 0.00 | 0.00 | 0.00 | 0.40 | 0.00 |
| *Canarium harveyi* Seem. | BURSERACEAE | 0.00 | 0.00 | 0.00 | 0.00 | 3.68 | 0.00 | 0.00 | 0.00 | 0.00 |
| *Cerbera manghas* L. | APOCYNACEAE | 3.68 | 0.00 | 0.00 | 0.00 | 0.00 | 0.00 | 0.00 | 0.00 | 0.00 |
| *Claoxylon fallax* Müll.Arg. | EUPHORBIACEAE | 0.00 | 0.00 | 0.19 | 0.00 | 0.00 | 0.78 | 0.00 | 0.00 | 0.00 |
| *Cocos nucifera* L. | ARECACEAE | 6.79 | 0.00 | 0.00 | 0.00 | 0.00 | 0.00 | 0.00 | 0.00 | 0.00 |
| *Cordyline fruticosa* (L.) A.Chev. | AGAVACEAE | 0.00 | 0.00 | 0.00 | 0.00 | 0.00 | 0.00 | 0.00 | 0.00 | 0.00 |
| *Cryptocarya hornei* Gillespie | LAURACEAE | 0.22 | 1.64 | 0.86 | 2.58 | 0.38 | 1.06 | 0.00 | 0.94 | 0.07 |
| *Cyclophyllum barbatum* (G.Forst.) A.C.Sm. & S.P.Darwin | RUBIACEAE | 0.00 | 0.00 | 0.00 | 0.00 | 0.00 | 0.00 | 0.00 | 0.05 | 0.00 |
| *Dendrocnide harveyi* (Seem.) Chew | URTICACEAE | 0.00 | 0.00 | 5.86 | 0.00 | 0.00 | 0.00 | 0.00 | 0.00 | 7.46 |
| *Diospyros elliptica* (J.R.Forst & G.Forst.) P.S.Green | EBENACEAE | 0.00 | 0.00 | 0.00 | 0.00 | 0.00 | 0.00 | 0.00 | 0.00 | 0.00 |
| *Diospyros samoensis* A.Gray | EBENACEAE | 0.00 | 0.00 | 0.00 | 0.00 | 0.00 | 0.00 | 0.00 | 0.00 | 0.00 |
| *Diospyros vitiensis* var. *longisepala* (Gillespie) A.C.Sm. | EBENACEAE | 0.00 | 0.00 | 0.00 | 0.00 | 0.00 | 0.10 | 0.00 | 0.14 | 0.00 |
| *Dysoxylum richii* (A.Gray) C.DC. | MELIACEAE | 7.95 | 0.00 | 1.53 | 11.62 | 7.35 | 0.19 | 0.04 | 0.10 | 13.06 |
| *Dysoxylum tenuiflorum* A.C.Sm. | MELIACEAE | 0.00 | 1.70 | 0.99 | 0.48 | 2.84 | 0.90 | 0.00 | 0.00 | 0.00 |
| *Elattostachys falcata* (A.Gray) Radlk. | SAPINDACEAE | 0.00 | 0.25 | 0.00 | 0.00 | 0.00 | 0.00 | 0.00 | 1.70 | 0.00 |
| *Erythrospermum acuminatissimum* (A.Gray) A.C.Sm. | FLACOURTIACEAE | 0.00 | 0.00 | 0.00 | 0.18 | 0.23 | 0.22 | 0.00 | 0.00 | 0.00 |
| *Eugenia reinwardtiana* (Blume) DC. | MYRTACEAE | 0.00 | 0.04 | 0.00 | 0.00 | 0.00 | 0.00 | 0.00 | 0.00 | 0.00 |
| *Ficus masonii* Horne ex Baker | MORACEAE | 0.00 | 0.00 | 0.00 | 0.00 | 0.00 | 0.00 | 0.00 | 0.00 | 0.00 |
| *Ficus obliqua* G.Forst. | MORACEAE | 5.65 | 0.00 | 0.00 | 0.00 | 0.00 | 0.00 | 0.00 | 0.00 | 6.01 |
| *Ficus prolixa* G.Forst. | MORACEAE | 0.00 | 0.00 | 0.00 | 0.00 | 0.00 | 0.00 | 0.00 | 0.00 | 0.00 |
| *Ficus scabra* G.Forst. | MORACEAE | 0.88 | 0.00 | 0.72 | 0.66 | 0.00 | 0.00 | 0.19 | 0.06 | 0.00 |
| *Flacourtia subintegra* A.C.Sm. | FLACOURTIACEAE | 0.00 | 0.00 | 0.00 | 0.00 | 0.00 | 0.14 | 0.00 | 1.76 | 0.00 |
| *Garcinia* *sessilis* (G.Forst.) Seem. | CLUSIACEAE | 0.00 | 0.00 | 0.25 | 0.00 | 0.92 | 0.12 | 0.00 | 0.00 | 0.00 |
| *Garuga floribunda* Decne. | BURSERACEAE | 0.00 | 0.00 | 0.00 | 0.00 | 0.00 | 0.35 | 0.00 | 0.00 | 5.84 |
| *Geniostoma rupestre* J.R.Forst. & G.Forst. | LOGANIACEAE | 0.20 | 0.00 | 0.00 | 0.00 | 0.00 | 0.00 | 0.00 | 0.00 | 0.00 |
| *Glochidion concolor* Müll.Arg. | EUPHORBIACEAE | 0.00 | 0.00 | 0.00 | 0.00 | 0.00 | 0.00 | 0.05 | 0.00 | 0.00 |
| *Guettarda speciosa* L. | RUBIACEAE | 0.00 | 0.00 | 0.13 | 0.00 | 0.00 | 0.00 | 0.00 | 0.00 | 0.00 |
| *Guioa rhoifolia* (A.Gray) Radlk. | SAPINDACEAE | 0.00 | 0.00 | 0.00 | 0.00 | 0.00 | 0.00 | 0.00 | 0.00 | 0.00 |
| *Gyrocarpus americanus* Jacq. | HERNANDIACEAE | 0.00 | 7.26 | 0.00 | 0.00 | 0.00 | 0.00 | 0.00 | 0.00 | 0.00 |
| *Harpullia arborea* (Blanco) Radlk. | SAPINDACEAE | 0.00 | 0.00 | 0.00 | 0.00 | 0.00 | 0.00 | 0.00 | 0.27 | 0.00 |
| *Heritiera ornithocephala* Kosterm. | STERCULIACEAE | 0.00 | 0.00 | 0.00 | 0.00 | 0.00 | 0.13 | 0.00 | 0.00 | 0.00 |
| *Hibiscus tiliaceus* L. | MALVACEAE | 2.35 | 0.00 | 0.00 | 0.00 | 0.00 | 0.00 | 0.00 | 0.00 | 0.00 |
| *Homalium pallidum* A.C.Sm. | FLACOURTIACEAE | 0.00 | 6.78 | 0.00 | 0.00 | 0.00 | 0.00 | 0.00 | 0.00 | 0.00 |
| *Intsia bijuga* (Colebr.) Kuntze | CAESALPINIACEAE | 0.00 | 0.00 | 0.00 | 0.00 | 0.00 | 0.88 | 0.00 | 0.00 | 0.00 |
| *Macaranga harveyana* (Müll.Arg.) Müll.Arg. | EUPHORBIACEAE | 0.00 | 0.00 | 0.57 | 0.00 | 0.00 | 0.00 | 0.51 | 0.00 | 0.00 |
| *Macaranga seemannii* (Müll.Arg.) Müll.Arg. | EUPHORBIACEAE | 0.00 | 0.00 | 0.00 | 0.00 | 0.00 | 0.00 | 7.52 | 7.65 | 0.14 |
| *Mangifera indica* L. | ANACARDIACEAE | 0.00 | 0.00 | 0.00 | 0.00 | 0.00 | 0.00 | 0.00 | 0.00 | 0.00 |
| *Manilkara vitiensis* (H.J.Lam & v.Olden) Meeuse | SAPOTACEAE | 0.00 | 0.00 | 0.00 | 0.51 | 0.43 | 0.15 | 0.00 | 0.00 | 0.00 |
| *Maniltoa floribunda* A.C.Sm. | CAESALPINIACEAE | 0.00 | 0.53 | 0.06 | 0.00 | 0.15 | 3.82 | 0.00 | 0.13 | 1.82 |
| *Melicope cucullata* (Gillespie) A.C.Sm. | RUTACEAE | 1.30 | 0.00 | 1.53 | 0.00 | 0.00 | 0.00 | 0.00 | 0.00 | 0.00 |
| *Melochia vitiensis* A.Gray | STERCULIACEAE | 0.00 | 0.00 | 0.00 | 0.00 | 0.00 | 0.00 | 0.00 | 0.00 | 0.00 |
| *Millettia pinnata* (L.) Panigrahi (syn. *Pongamia pinnata* (L.) Pierre) | FABACEAE | 1.32 | 2.02 | 0.00 | 0.00 | 0.00 | 0.20 | 0.00 | 0.00 | 0.00 |
| *Myristica* *gillespieana* A.C.Sm. | MYRISTICACEAE | 0.00 | 0.31 | 6.08 | 0.00 | 0.04 | 0.00 | 1.39 | 0.00 | 0.77 |
| *Neisosperma oppositifolia* (Lam.) Fosberg & Sachet | APOCYNACEAE | 0.00 | 0.00 | 0.11 | 0.43 | 0.00 | 0.00 | 0.00 | 0.00 | 0.00 |
| *Neonauclea forsteri* (Seem. ex Havil.) Merr. | RUBIACEAE | 0.00 | 0.00 | 0.00 | 0.00 | 11.16 | 0.00 | 0.00 | 0.00 | 0.00 |
| *Pandanus tectorius* Parkinson | PANDANACEAE | 0.00 | 0.00 | 0.00 | 0.00 | 0.00 | 0.00 | 0.00 | 0.00 | 0.00 |
| *Phaleria pubiflora* (A.Gray) Gilg | THYMELAEACEAE | 0.00 | 0.00 | 0.00 | 0.00 | 0.00 | 0.00 | 0.00 | 0.00 | 0.00 |
| *Pisonia grandis* R.Br. | NYCTAGINACEAE | 0.00 | 1.09 | 0.00 | 0.00 | 0.00 | 0.00 | 0.00 | 0.00 | 0.00 |
| *Pittosporum arborescens* Rich ex A.Gray | PITTOSPORACEAE | 0.00 | 0.41 | 0.00 | 0.00 | 0.00 | 0.00 | 0.00 | 0.00 | 1.24 |
| *Pleiogynium timoriense* (DC.) Leenh. | ANACARDIACEAE | 0.00 | 0.00 | 0.09 | 2.71 | 2.15 | 6.81 | 0.00 | 0.00 | 17.71 |
| *Polyalthia laddiana* A.C.Sm. | ANNONACEAE | 0.00 | 0.00 | 0.00 | 0.00 | 0.00 | 0.00 | 0.00 | 0.00 | 0.00 |
| *Polyalthia vitiensis* Seem. | ANNONACEAE | 0.00 | 0.00 | 0.00 | 0.00 | 0.00 | 0.00 | 0.00 | 1.90 | 0.00 |
| *Polyscias multijuga* (A.Gray) Harms | ARALIACEAE | 0.63 | 0.27 | 0.00 | 0.00 | 0.00 | 0.00 | 0.00 | 0.11 | 0.00 |
| *Pometia pinnata* J.R.Forst. & G.Forst. | SAPINDACEAE | 0.00 | 0.00 | 0.00 | 0.00 | 6.37 | 0.00 | 0.00 | 0.00 | 0.00 |
| *Pouteria grayana* (H.St.John) Fosberg (syn. *Planchonella grayana* H.St.John) | SAPOTACEAE | 0.24 | 0.21 | 0.00 | 0.63 | 0.00 | 0.00 | 0.00 | 0.00 | 1.04 |
| *Premna serratifolia* L. | VERBENACEAE | 0.00 | 0.00 | 0.00 | 0.00 | 0.00 | 0.00 | 0.06 | 3.91 | 0.00 |
| *Serianthes melanesica* Fosberg | MIMOSACEAE | 0.00 | 0.00 | 0.00 | 0.00 | 0.00 | 0.00 | 0.00 | 1.75 | 0.00 |
| *Syzygium* aff. g*racilipes (undescribed)* | MYRTACEAE | 0.00 | 0.00 | 0.00 | 0.00 | 0.00 | 0.00 | 0.00 | 0.00 | 0.00 |
| *Syzygium quadrangulatum* (A.Gray) Merr. & L.M.Perry | MYRTACEAE | 0.00 | 0.00 | 0.35 | 0.00 | 0.00 | 0.00 | 0.00 | 0.00 | 0.21 |
| *Syzygium richii* (A.Gray) Merr. & L.M.Perry | MYRTACEAE | 0.00 | 0.00 | 0.00 | 0.00 | 0.36 | 0.00 | 0.00 | 0.07 | 1.10 |
| *Syzygium seemannii* (A.Gray) Biffin & Craven | MYRTACEAE | 0.00 | 0.00 | 0.00 | 0.00 | 0.00 | 0.29 | 0.00 | 0.00 | 0.00 |
| *Syzgium* sp. Nova (undescribed) | MYRTACEAE | 0.54 | 0.00 | 0.00 | 0.00 | 0.00 | 0.00 | 0.00 | 0.00 | 0.00 |
| *Tabernaemontana pandacaqui* Lam. (syn. *Ervatamia obtusiuscula* Markgr.) | APOCYNACEAE | 0.00 | 0.19 | 0.35 | 0.00 | 0.00 | 0.00 | 0.00 | 0.13 | 0.25 |
| *Terminalia catappa* L. | COMBRETACEAE | 0.00 | 0.00 | 0.00 | 0.00 | 0.00 | 0.00 | 0.00 | 0.00 | 0.00 |
| *Vavaea amicorum* Benth. | MELIACEAE | 0.06 | 0.00 | 0.00 | 0.00 | 0.00 | 0.47 | 0.00 | 0.49 | 0.04 |
| *Xylosma simulans* A.C.Sm. | FLACOURTIACEAE | 0.34 | 0.40 | 0.00 | 0.00 | 0.00 | 0.00 | 0.00 | 0.00 | 0.00 |
| Indeterminate Flacourtiaceae 41 | FLACOURTIACEAE | 0.00 | 0.00 | 0.94 | 0.00 | 0.00 | 0.00 | 0.00 | 0.00 | 0.00 |
| Indeterminate Lauraceae 20-16 | LAURACEAE | 0.00 | 0.00 | 0.00 | 0.00 | 0.00 | 0.20 | 0.00 | 0.00 | 0.00 |
| Indeterminate Lauraceae 21-5 | LAURACEAE | 0.00 | 0.00 | 0.00 | 0.00 | 0.00 | 0.00 | 16.28 | 0.00 | 0.00 |
| Indeterminate Lauraceae cf. Litsea sp. | LAURACEAE | 0.00 | 0.00 | 0.00 | 0.00 | 0.00 | 2.57 | 0.00 | 0.45 | 0.00 |
| Indeterminate Pisonia sp. | NYCTAGINACEAE | 0.00 | 1.09 | 0.00 | 0.00 | 0.00 | 0.00 | 0.00 | 0.00 | 0.00 |
| Indeterminate Syzygium sp. | MYRTACEAE | 0.00 | 0.00 | 0.00 | 0.00 | 0.00 | 0.00 | 0.00 | 0.00 | 0.00 |
| Indeterminate family 17-15 | unknown | 0.00 | 0.00 | 3.18 | 0.00 | 0.00 | 0.00 | 0.00 | 0.00 | 0.00 |
| Indeterminate family 18-3 | unknown | 0.00 | 0.00 | 0.00 | 0.83 | 0.00 | 0.00 | 0.00 | 0.00 | 0.00 |
| Forest Group | unitless, 1-4 (see text) | 2 | 3 | 2 | 3 | 2 | 2 | 2 | 2 | 2 |
| Slope | degrees | 0 | 17 | 30 | 0 | 0 | 0 | 0 | 10 | 20 |
| Aspect | degrees | 0.1 | 240 | 180 | 0.1 | 0.1 | 0.1 | 0.1 | 90 | 270 |
| Elevation | meters | 10 | 35 | 50 | 30 | 150 | 120 | 135 | 140 | 65 |
| Rockiness | percent cover | 0 | 100 | 50 | 30 | 20 | 70 | 0 | 90 | 30 |
| Canopy Height | meters | 15 | 12 | 17 | 18 | 25 | 22 | 25 | 20 | 20 |
| Canopy cover | percent cover | 80 | 80 | 90 | 80 | 80 | 80 | 80 | 90 | 80 |
| Total Basal Area | square meters per ha | 53.02 | 25.02 | 24.61 | 32.36 | 49.31 | 31.80 | 34.43 | 26.08 | 62.39 |
